# Peer support for people with chronic conditions: a systematic review of reviews

**DOI:** 10.1186/s12913-022-07816-7

**Published:** 2022-03-31

**Authors:** Dean M. Thompson, Lesley Booth, David Moore, Jonathan Mathers

**Affiliations:** 1 Institute of Applied Health Research, University of Birmingham, Birmingham, UK; 2 Bowel Research UK, London, UK

**Keywords:** Chronic conditions, Peer support, Review of reviews, Systematic review

## Abstract

**Background:**

People with chronic conditions experience functional impairment, lower quality of life, and greater economic hardship and poverty. Social isolation and loneliness are common for people with chronic conditions, with multiple co-occurring chronic conditions predicting an increased risk of loneliness. Peer support is a socially driven intervention involving people with lived experience of a condition helping others to manage the same condition, potentially offering a sense of connectedness and purpose, and experiential knowledge to manage disease. However, it is unclear what outcomes are important to patients across the spectrum of chronic conditions, what works and for whom. The aims of this review were to (1) collate peer support intervention components, (2) collate the outcome domains used to evaluate peer support, (3) synthesise evidence of effectiveness, and (4) identify the mechanisms of effect, for people with chronic conditions.

**Methods:**

A systematic review of reviews was conducted. Reviews were included if they reported on formal peer support between adults or children with one or more chronic condition. Data were analysed using narrative synthesis.

**Results:**

The search identified 6222 unique publications. Thirty-one publications were eligible for inclusion. Components of peer support were organised into nine categories: social support, psychological support, practical support, empowerment, condition monitoring and treatment adherence, informational support, behavioural change, encouragement and motivation, and physical training. Fifty-five outcome domains were identified. Quality of life, and self-efficacy were the most measured outcome domains identified. Most reviews reported positive but non-significant effects.

**Conclusions:**

The effectiveness of peer support is unclear and there are inconsistencies in how peers are defined, a lack of clarity in research design and intervention reporting, and widely variable outcome measurement. This review presents a range of components of peer support interventions that may be of interest to clinicians developing new support programmes. However, it is unclear precisely what components to use and with whom. Therefore, implementation of support in different clinical settings may benefit from participatory action research so that services may reflect local need.

**Supplementary Information:**

The online version contains supplementary material available at 10.1186/s12913-022-07816-7.

## Introduction

Noncommunicable diseases or chronic conditions are illnesses, which typically persist for 12 months or more, resulting in functional impairments involving some limitation in social, occupational, or other sphere of life due to illness, requiring health or social care intervention [1, 2]. These include, though are not limited to cardiovascular disease, respiratory disease, cancer and diabetes. Global premature avertable mortality across 43 chronic conditions is estimated at 9008 years of life lost per 100,000 population [3], and the prevalence of chronic conditions and multimorbidity is increasing in the twenty-first century [4].

Chronic conditions, for example diabetes, affects labour market participation with greater absence, unemployment, early retirement and disability pension [5], with total health care expenditure estimated at $727 billion globally [6]. Approximately 12-18% of NHS expenditure is directed towards chronic conditions associated with poor mental health [7]. People with chronic conditions experience lower quality of life [8], and greater economic hardship and poverty [9]. A sense of isolation, alienation and loneliness are common to people with chronic conditions [10], with multiple co-occurring chronic conditions predicting an increased risk of loneliness [11]. Loneliness is associated with a poorer course of disease progression and predicts treatment outcomes [12, 13].

Progress in addressing chronic conditions has been inconsistent, with government policy insufficient to scale back the epidemic of disease [14]. In the US, people with chronic conditions without health insurance are less likely than those with health insurance to visit a healthcare professional [15], and the burden of disease is greatest in low- and middle-income countries where health and social care provision is scarce [16]. The World Health Organization (WHO) global action plan on noncommunicable disease relies on the overarching principle of empowerment of people and communities through participation in grass-roots organisations and the provision of tools to enable self-management [17]. People with chronic conditions spend as much as 2 h per day on self-management of their health [18]. Much of this effort is informally supported by family who may lack experiential knowledge to offer holistic support while also being at heightened risk of experiencing emotional, social, physical and financial burden themselves [19–21]. Formal peer support interventions represent one potential solution to empower people to manage their own health, while reducing burden on informal carers and healthcare systems. Peer support involves people with lived experience of a condition helping others to manage the same condition, potentially offering a sense of connectedness and purpose, and experiential knowledge to manage disease. The introduction of formal peer support interventions such as the Stanford Chronic Disease Self-Management Program (CDSMP) and the Expert Patient Programme (EPP) offers potential for economies of scale and cost savings for healthcare services [22–24]. Major chronic conditions share common modifiable risk factors including unhealthy diet, physical inactivity, tobacco use, and harmful alcohol use, which feed into intermediate risk factors including raised blood pressure and blood glucose, abnormal blood lipids and obesity. Yet most reviews of peer support focus on one chronic condition in isolation, and there are few reviews on peer support that cut across chronic conditions. Those that do are limited to online peer support [25, 26], peer support specifically delivered in rural areas and other hard to reach populations [27, 28], peer support for adolescent populations [29], peer support focused on chronic pain only [30], and meta-ethnography of qualitative research focused on self-reported experience of peer support [10].

It is unclear what outcomes are important across the spectrum of chronic conditions, what works and for whom. These uncertainties impede healthcare services and charitable organisations from developing, implementing and evaluating peer support interventions. As the WHO 2013-2020 global action plan draws to an end with limited progress in preventing and controlling noncommunicable disease, a systematic review of reviews on peer support summarising and comparing conclusions across chronic conditions is timely.

The aims of this review of peer support in chronic conditions were to (1) describe peer support intervention components, (2) identify the outcome domains that have been used to evaluate peer support, (3) summarise evidence of effectiveness, and (4) identify mechanisms of effect that have been proposed for peer support interventions.

## Methods

This review was written in accordance with PRISMA guidance (see Supplementary Material 1 for the PRISMA checklist).

### Protocol registration

A systematic review of reviews protocol was written with reference to PRISMA guidelines [31], and registered on PROSPERO (International Prospective Register of Systematic Reviews) on 25 September 2019: CRD42019127906.

### Eligibility criteria

Study eligibility criteria were developed using the PICOS (Population, Intervention, Comparison, Outcome, and Study design) framework. We included adults and children with one or more chronic condition, defined as a disease or illness, which typically persists for 12 months or more, resulting in functional impairments requiring health or social care intervention [1, 2]. We included any peer support intervention, defined here as any formal support provided and received by people with a shared experience of having a chronic condition. ‘Formal’ peer support refers to interventions arranged by organisations rather than spontaneous peer support between individuals or groups in their day-to-day environment. No reviews were excluded on the basis of outcomes, comparators or control conditions. Any type of review listed in Grant and Booth’s (2009) typology of reviews were included [32]. We included each of these review types to be inclusive and comprehensive in identifying intervention components. We excluded reviews where peer support interventions were not the primary focus of the review or where the intervention, outcome domain, effectiveness and mechanism data for peer support interventions could not be delineated from other types of interventions. Reviews that combined eligible and ineligible types of ‘peers’ were included if the intervention, outcome domain, effectiveness or mechanism data could be delineated by definition of ‘peer’.

### Information sources

Six data sources were used: (1) electronic bibliographic databases (*n* = 9) were searched on 30th September 2019 and updated on 30th July 2020, including MEDLINE, EMBASE, PsycINFO, CINAHL, Scopus, Web of Science, Cochrane Database of Systematic Reviews, Google Scholar, and ProQuest Dissertations & Theses Global; (2) PROSPERO was searched for ongoing systematic reviews and corresponding authors contacted for unpublished manuscripts; (3) one website was hand-searched (http://peersforprogress.org/); (4) forward citation tracking of included publications via Scopus, (5) backward citation tracking of included publications by hand-searching reference lists was performed; and (6) a preliminary list of included publications was sent to experts (*n* = 53; authors of included reviews) requesting additional eligible publications.

### Search

The search strategy combined terms for peer support with terms for chronic conditions. Peer support search terms were adapted from a published systematic review concerning peer support [33] and peer reviewed by an expert in literature search design. Terms for chronic conditions encompassed those conditions representing greater than 1% of global Disability Adjusted Life Years (DALYs) according to the World Health Organisation estimates (2018) [34], were specified in the search. The search strategy was tailored to each electronic bibliographic database and where available and possible used index terms in addition to free text terms (see Supplementary Material 2 for the search strategy used for MEDLINE).

### Study selection

Citations were exported into Clarivate Analytics’ EndNote X9 software [35], and duplicates were removed by using the ‘find duplicate’ function and manually hand-searching the list of publications. DT screened the title and abstract of each identified publication against the inclusion criteria. A randomly selected sample of 10% of title and abstracts were independently assessed by two reviewers (concordance = 91%). The full text of each publication was screened by DT. A randomly selected sample of 10% of full texts were independently assessed by two reviewers (concordance = 91%).

### Data items

For each publication, we extracted data on: (1) review information including the type of review, review aims, definition of peer support used in the reviews, review eligibility criteria, primary research design, primary research comparators, date of searches and review risk of bias appraisal tool and appraisal findings; (2) setting of primary research; (3) participants of primary research, including demographic and clinical characteristics; (4) the peer support programmes used in primary research (adapted from [36, 37]; (5) outcome measurement domains used in primary research; (6) a narrative summary of reviews’ findings on effectiveness (effect sizes and confidence intervals are reported where these have been systematically reported; and (7) theories and mechanisms of effect reported in the findings of included publications.

DT and JM independently piloted the data extraction table with a sample of 5 publications. DT and JM or MG discussed their data extraction (concordance = 85%). Discrepancies were identified and resolved through discussion and the data extraction table instructions were refined until agreement in the pilot sample was greater than 90% (with no data extraction omissions by the lead reviewer, DT) before DT completed data extraction.

### Risk of bias in individual reviews

Included reviews underwent critical appraisal using AMSTAR 2 [37]. AMSTAR 2 is a 16-item measurement tool to assess systematic reviews, though was applied to other types of reviews to enable a best evidence synthesis. DT assessed each included review against each of the 16 items. A randomly selected sample of 10% of included reviews were independently assessed along with all other data items as described in the process above (concordance = 85%). Each review was organised into AMSTAR 2 categories including critically low, low, moderate, and high overall confidence in the results of the review [38].

### Synthesis of results

A 3-stage narrative synthesis was conducted based on Popay and colleagues’ (2006) guidance [39]. Stage 1 involved developing a preliminary synthesis. Intervention components, outcome domains, and mechanisms of effect in peer support interventions were tabulated and an initial framework was developed using simple content analysis to group related data [40]. Vote counting was performed to determine the number of reviews identifying each intervention component, outcome domain, and mechanism. The identified components, outcomes, and mechanisms were iteratively grouped from the bottom-up using the extracted data. The effectiveness of the peer support interventions was tabulated for the outcome domains most frequently reported by the included reviews. Pooled effect sizes from the included reviews were reported here where available. Stage 2 involved exploring relationships between studies. Effectiveness data and mechanisms of effect are presented according to chronic condition. Stage 3 involved assessing the robustness of the synthesis. A best evidence synthesis of intervention components, outcome domains, effectiveness, and mechanisms of effect in high quality reviews (based on AMSTAR2) was planned.

## Results

### Study selection

**Fig. 1:**
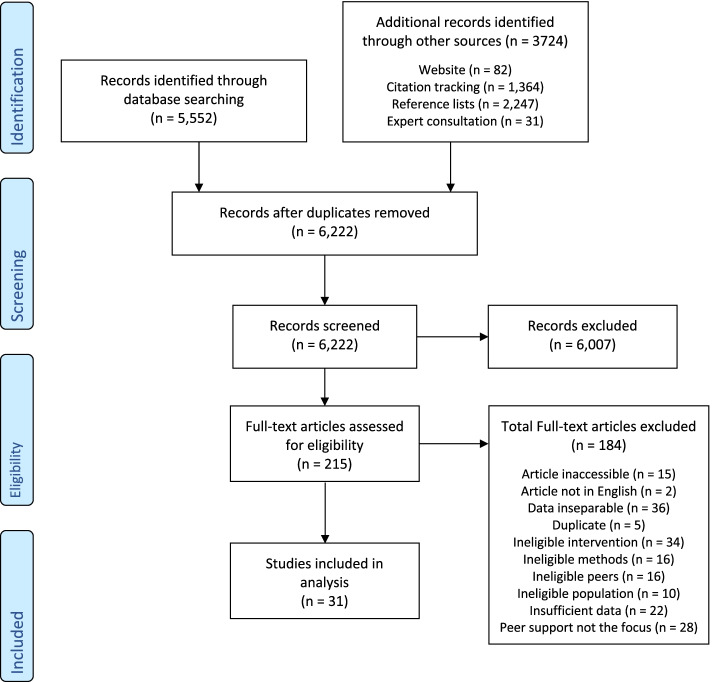
Flow diagram of study selection

The search identified 6222 unique publications (Fig. 1). Of the 215 screened full-texts, 184 were excluded (see Supplementary Material 3 for list of and reasons for excluding each publication from electronic bibliographic databases). Thirty-one publications were eligible for inclusion [10, 25, 27, 36, 37, 41–66].

### Developing a preliminary synthesis and exploring relationships between studies

#### Study characteristics

**Fig. 2:**
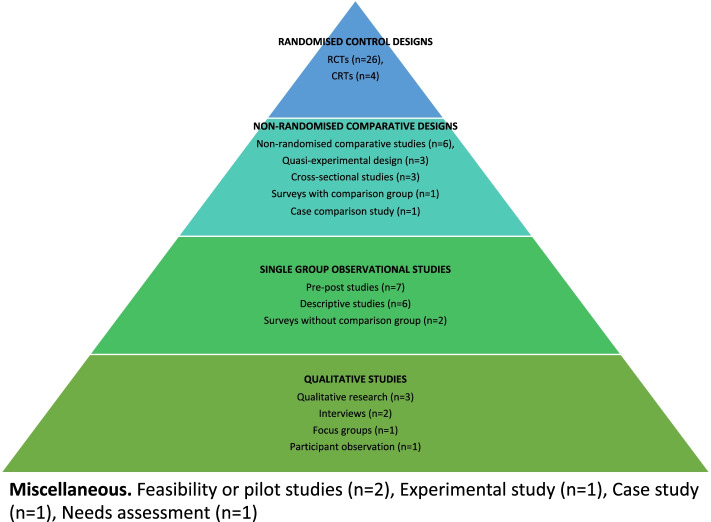
Peer support primary research study designs

Reported review types include systematic reviews (*n* = 14), literature reviews (*n* = 6), reviews including meta-analysis (*n =* 6), scoping reviews (*n* = 3), and qualitative evidence syntheses (*n* = 2). The primary research designs reported in the reviews were organised into 4 tiers (Fig. 2), including Randomised control designs (number of reviews including this desig*n* = 26), Non-randomised comparative designs (*n* = 13), Single group observational studies (*n* = 12), and Qualitative studies (*n* = 5). Randomised controlled designs included RCTs (*n =* 26), and Cluster Randomised Trials (CRTs) (*n* = 4; see Supplementary Material 4 for study datasets). Non-randomised comparative designs included non-randomised comparative studies (*n* = 6), quasi-experimental design (*n* = 3), cross-sectional studies (*n =* 3), a survey with comparison group (*n* = 1), and a case comparison study (*n =* 1). Single group observational studies included pre-post studies (*n* = 7), descriptive studies (*n* = 6), and surveys without comparison group (*n* = 2). Qualitative studies included unspecified qualitative research (*n* = 3), interviews (*n =* 2), focus groups (*n* = 1), and participant observation (*n* = 1). Some reviews reported primary research designs which could not be categorised or did not appear to be evaluative including feasibility or pilot studies (*n* = 2), an experimental study (*n* = 1), a case study (*n =* 1), and a needs assessment (*n* = 1).

**Fig. 3:**
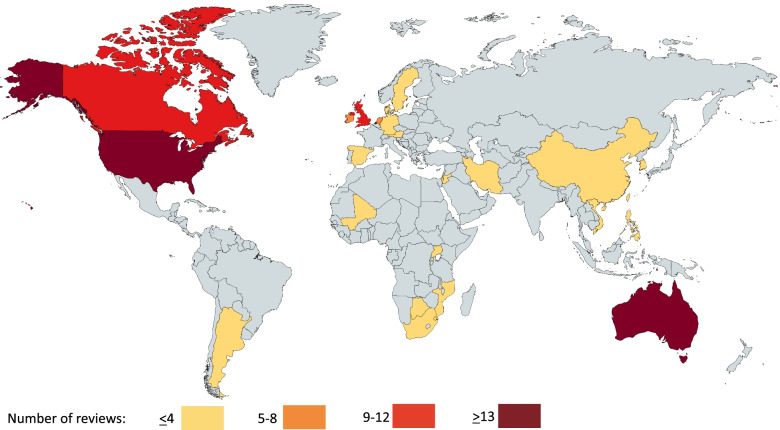
Peer support primary research settings

The conditions reviewed included cancer (*n* = 9), diabetes (*n* = 7), cardiovascular disease (*n* = 4), acquired brain injury, cerebral palsy, and spina bifida (*n* = 1), asthma (*n =* 1), kidney disease (*n =* 1), HIV (*n =* 1), and somatic illness (*n =* 1). Six reviews included any chronic condition. The reviews included primary research across 26 countries or semi-autonomous regions (Fig. 3). Reviews reported on primary research based in USA (*n =* 24), Australia (*n =* 14), Canada (*n =* 12), the UK (*n* = 11), Republic of Ireland (*n* = 6), Netherlands (*n* = 4), Argentina (*n* = 3), China, Germany, Mozambique, South Korea, Sweden, Uganda, and Vietnam (*n* = 2); Austria, Botswana, Denmark, Hong Kong, Iran, Israel, Jordan, Mali, Philippines, South Africa, Spain, and Taiwan (*n* = 1). Reviews reported on primary research based in community settings (*n* = 8), general hospitals (*n* = 5), outpatient clinics (*n* = 4), HIV/AIDS clinics (*n* = 3), school or other educational settings (*n =* 3), US Veterans Affairs centres (*n =* 3), camps (*n* = 2), primary care settings (*n =* 2), church (*n* = 1), haemodialysis centre (*n* = 1), HIV/AIDS clinical trials unit (*n* = 1), infectious disease hospital unit (*n* = 1), physiotherapy department (*n =* 1), public health clinic (*n =* 1), seniors’ centre (*n =* 1), stroke rehabilitation centre (*n =* 1), University clinic (*n =* 1), workplace (*n =* 1).

#### Peer support intervention description

Reviews reported on peer support delivered in groups (*n* = 21) and one-to-one (*n* = 17), by telephone (*n =* 21), face-to-face (*n* = 16), and online (*n* = 13). Descriptions of peer support intervention components most frequently comprised education (*n =* 13), self-management techniques (*n* = 9), discussion (*n* = 7), reciprocal support (*n =* 7), sharing personal experiences (*n* = 6), and unspecified social support (*n =* 6). Other specific forms of social support were numerous, wide ranging, and are listed in full in Table 1. Peers were consistently defined by their role in providing or exchanging support between people with similar experiences or circumstances, though only seven specified that this similarity extends to first-hand experience of living with a shared chronic condition, and three reviews did not give a definition of what constitutes peer support. The duration of peer support interventions ranged from a single session to 2 years. Most reviews reported on interventions with one contact per week, with a range of daily (for up to 1 week) to 3- and 12-monthly follow-ups.

**Table 1:** Intervention description table

Intervention components	Number of reviews
Education	13 (42%)
Self-management	9 (29%)
Discussion	7 (23%)
Reciprocally giving support to others	7 (23%)
Sharing personal experiences	6 (19%)
Unspecified social support	6 (19%)
Medications advice/ adherence	5 (16%)
Emotional support	4 (13%)
Activity scheduling/ planning leisure activities	3 (10%)
Addressing unspecified psychosocial issues	3 (10%)
Cognitive techniques	3 (10%)
Community outreach	3 (10%)
Encouragement	3 (10%)
Goal setting	3 (10%)
Mentoring	3 (10%)
Modelled recovery	3 (10%)
Addressing physical health concerns	2 (6%)
Counselling	2 (6%)
Exercise	2 (6%)
Monitoring condition/ symptoms	2 (6%)
Psychoeducation	2 (6%)
Strategic thinking	2 (6%)
Talking Circles (native American cultural discussion facilitation)	2 (6%)
Unspecified psychological support	2 (6%)
Answered patient questions	1 (3%)
Bullying support	1 (3%)
Coping skills	1 (3%)
Decision making	1 (3%)
Developing relationships	1 (3%)
Emphasise personal achievement	1 (3%)
Encouraging contact with clinicians	1 (3%)
Lifestyle change exercises	1 (3%)
Low-level advice	1 (3%)
Lead group activities	1 (3%)
Mindfulness	1 (3%)
Motivational support	1 (3%)
Needs assessment	1 (3%)
Outings for social integration and networking	1 (3%)
Problem-solving	1 (3%)
Recognising trauma	1 (3%)
Relaxation techniques	1 (3%)
Smoking cessation counselling	1 (3%)
Unspecified behavioural change	1 (3%)
Unspecified practical support	1 (3%)
Ways of taking action	1 (3%)

Peer training content and methods were organised into 11 categories including delivery methods, counselling skills, communication skills, condition and treatment information and adherence, meta-competency and safety, social skills and story sharing, intervention and pedagogical theory, culture and religion, physical training techniques, intervention facilitation skills, and availability of community resources. Training most frequently involved teaching communication skills (*n* = 7), condition and treatment information (*n* = 5; see Supplementary Material 5 for training description table). Five reviews reported whether primary research peer support included the use of an intervention manual.

Eleven reviews reported on the supervision of peer support. Three reviews simply indicated whether supervision was a component of the primary research intervention, and 2 reviews indicated whether supervision was professionally delivered. Six reviews described supervision in more detail including supervision of the first session only (*n* = 1), weekly supervision from a psychologist, nurse, and community outreach coordinator (*n* = 1), a study coordinator called participants to identify problems, including problems with the peer support relationship at week 3 of the intervention (*n* = 1), an endocrinologist, an exercise physiologist and an exercise health psychologist were available to answer questions throughout the study period (*n* = 1), psychologist supervision (*n =* 1), nurse supervision (*n =* 1), facilitators were present to ensure fidelity to the research protocol (*n =* 1), children and young people overseen by adults (*n* = 1).

Four reviews reported on screening practices to recruit peer support workers based on success (e.g. reduction in severity of condition or improved self-management) from past treatment (*n* = 1), previous experience facilitating a group, ability to motivate, good listening and problem-solving skills (*n* = 1), knowledge of diabetes and an interest in helping people and effective communication patterns (*n* = 1), a competency test involving role play (*n* = 1), severity of chronic condition (*n =* 1); from the same community (*n* = 1), demonstration of leadership qualities (*n =* 1), ability to engage in conversation (*n =* 1), give information clearly (*n =* 1), share experiences and display appropriate listening skills (*n =* 1). Twelve reviews reported on peer matching including matching by age (*n* = 5), ethnicity (*n* = 2), cultural similarities (*n =* 2), type of cancer (*n =* 2), life factors (*n* = 1), gender (*n =* 1), primary language (*n =* 1), men who have sex with men (*n =* 1), chronic pain (*n =* 1), injection drug use (*n =* 1), ex-smoker status (*n =* 1).

#### Outcome domains

Fifty-five outcome domains were identified and organised across 15 categories (Table 2). Mental health and psychosocial processes and adjustment were the most frequently reported outcome categories. Quality of life (*n* = 14), self-efficacy (*n =* 14), clinical surrogates (*n* = 12), depression (*n* = 11), distress (n = 11), and health knowledge (*n* = 9) were the most frequently analysed outcome domains in reviews. Psychosocial process and adjustment was the most variably measured category, spanning 21 outcome domains.

**Table 2:** Outcome domains identified in reviews

Domains	Number of reviews
**Mental health**	**18 (58%)**
Depression	11 (35%)
Distress	11 (35%)
Anxiety	7 (23%)
Mental health	5 (16%)
Post-traumatic stress	2 (6%)
Wellbeing	2 (6%)
Suicidal ideation	1 (3%)
**Psychosocial processes & adjustment**	**16 (52%)**
Self-efficacy/ confidence	*14 (45%)*
Optimism/ Pessimism/ Hope	4 (13%)
Coping	3 (10%)
Empowerment	3 (10%)
Social coping	3 (10%)
Adjustment	2 (6%)
Psychosexual functioning	2 (6%)
Altruism	1 (3%)
Catharsis	1 (3%)
Comfort with clinician	1 (3%)
Illness uncertainty	1 (3%)
Motivation to volunteer	1 (3%)
Negative affect	1 (3%)
Perceived threat of condition	1 (3%)
Positive upward comparison	1 (3%)
Post-traumatic growth	1 (3%)
Self-understanding	1 (3%)
Sense of coherence	1 (3%)
Spirituality	1 (3%)
Stigma	1 (3%)
Suppression of affect	1 (3%)
**Quality of life**	**14 (45%)**
**Clinical surrogate**^a^	**12 (39%)**
**Physical health & function**	**12 (39%)**
Functional status	5 (16%)
Health status	4 (13%)
Physical health	4 (13%)
Adverse events	1 (3%)
**Social integration & connectedness**	**12 (39%)**
Social support	8 (26%)
Connectedness/ social network	3 (10%)
Social isolation	3 (10%)
Acculturation	1 (3%)
Community integration	1 (3%)
Interpersonal relationships	1 (3%)
**Health knowledge**	**9 (29%)**
**Health care utilisation**	**9 (29%)**
**Health behaviour**	**8 (26%)**
**Self-care**	**8 (26%)**
**Treatment adherence**	**8 (26%)**
**Symptom severity**	**6 (19%)**
Condition symptom severity	5 (16%)
Pain severity	2 (6%)
**General level of activity**	**4 (13%)**
**Quality of communication with others**	**3 (10%)**
**Mortality**	**1 (3%)**

^a^Clinical surrogates: Blood glucose (Fasting blood glucose); Blood pressure (Diastolic blood pressure & Systolic blood pressure); CD4 cell count; Estimated Glomerular Filtration Rate (eGFR); Glycated haemoglobin (HbA1c); Lipid Levels/ Lipid Profile (High-density lipoproteins & Low-density lipoproteins); Prostate-specific antigen; Resting heart rate; Triglyceride; Urinalysis (Glycosuria, Microhematuria, or Proteinuria); Viral Load; Weight (Body fat, Waist circumference, or Body Mass Index)

#### Effectiveness

For the most frequently identified outcome domains (*n* = 6), the majority reviews that were designed to evaluate effectiveness and reported pooled effect sizes and confidence intervals reported mostly statistically non-significant effects of peer support (Table 3). Though most reviews reported findings favouring peer support, Table 3 shows that these were significant only in reviews of cardiovascular disease and diabetes measuring HBA1c and blood pressure. Specifically, three meta-analyses pooled data on clinical surrogates, indicating small to medium statistically significant differences in favour of peer support effect sizes for HBA1c [55, 64, 65]. Effect sizes were small though not statistically significant for quality of life and depression [53, 59], and negligible and not statistically significant for distress [54].

**Table 3:** Effect size (MD) of peer support interventions (or number of significant findings of primary studies)

	Quality of life	Self-efficacy	Clinical surrogates	Depression	Distress	Health knowledge
**Asthma**						
Kew 2017	*n =* 3, 0.40 (95% CI − 0.02 to 0.81) favouring peer support					
**Cancer**						
McCaughan 2017	*n =* 3, −0.11 (95% CI − 0.47 to 0.24) favouring peer support			*n =* 5, −0.37 (95% CI − 0.75 to 0.00) favouring peer support		
**Cardiovascular disease**						
Small 2013			HBA1c *n =* 4, −0.26 (95% CI − 0.41 to − 0.11, I^2^ = 47.6% favouring peer support; Blood pressure *n =* 1, − 0.25 (95% CI − 0.45 to − 0.05) favouring peer support			
**Diabetes**						
Kong 2020					*n =* 10, −0.06 (95% CI − 0.22 to 0.10) favouring peer support	
Krishnamoorthy 2018			HBA1c *n =* 26, −0.28 (95% CI − 0.45 to − 0.11) favouring peer support			
Qi 2015			HBA1c *n =* 13, −0.57 (95% CI − 0.78 to − 0.36) favouring peer support			

#### Mechanisms

Twenty-three mechanisms or theories were identified across 9 reviews (Table 4). Most mechanisms or theories were identified in two reviews that focused on theory and treatment experience [10, 42]. Most reviews did not aim to address intervention theory. Appraisal, emotional and informational social support (*n* = 5), social cognitive theory (*n* = 3), and social comparison theory (*n =* 3) were the most frequently cited mechanisms or theories of effect in peer support interventions.

**Table 4:** Mechanisms identified in reviews

	Social cognitive theory	IMB Theory	Uncertainty management theory	Appraisal, emotional, informational support	Illness or Social identity theory	Readiness stage of wellness motivation	Theory of planned behaviour	Empowerment	Peer leadership or advocacy	Sense of connection	Experiential knowledge
**Cancer**											
Dunn 2003											
Lee 2018	✓								✓		
Meyer 2015											
Walshe 2018				✓							
**Chronic disease**											
Embuldeniya 2013										✓	✓
Enriquez 2016		✓		✓							
**Diabetes**											
Qi 2015	✓			✓				✓			
**HIV**											
Boucher 2020	✓	✓	✓	✓	✓	✓	✓	✓	✓		
**Somatic illness**											
Kingod 2017				✓	✓						✓

	Finding meaning	Isolation	Sharing	Helping	Reciprocity	Role satisfaction	Emotional entanglement	Self-determination & autonomy motivation	Social comparison theory	Orem’s self-care theory	Rodger’s person-centred approach	Collective voice & mobilisation
**Cancer**												
Dunn 2003									✓			
Lee 2018												
Meyer 2015									✓			
Walshe 2018									✓			
**Chronic disease**												
Embuldeniya 2013	✓	✓	✓	✓	✓	✓	✓					
Enriquez 2016								✓		✓	✓	
**Diabetes**												
Qi 2015								✓				
**HIV**												
Boucher 2020												
**Somatic illness**												
Kingod 2017												✓

IMB Theory (Information, motivation, and behavioural skills health behaviour model); Biopsychosocial ICF theoretical framework (Biopsychosocial International Classification of Functioning, Disability and Health theoretical framework)

#### Assessing the robustness of the synthesis

Overall confidence in reviews were rated high in two Cochrane reviews [46, 53], low (*n* = 5) and critically low (*n* = 24). Most reviews were rated ‘critically low’ due to 2 recurring critical weaknesses: absence of explicit reference to a protocol established prior to the conduct of the review (*n* = 16), and the absence of reference to a list of excluded studies and justification for exclusions (*n* = 18; Table 5). However, each of these reviews did not necessarily set out to be read as a comprehensive systematic review. Of the reviews that assessed risk of bias, most used the Cochrane risk of bias tool (*n* = 13), and a further three referred to the EPOC-specific Cochrane risk of bias tool [67]. Reviews’ risk of bias findings were rated favourably with low risk of bias and high confidence in the findings (regarding cancer, cardiovascular disease, and somatic illness; *n* = 5); equivocal or unclear (*n* = 6); and rated unfavourably with high risk of bias and low confidence in the findings (*n* = 8).

**Table 5:**
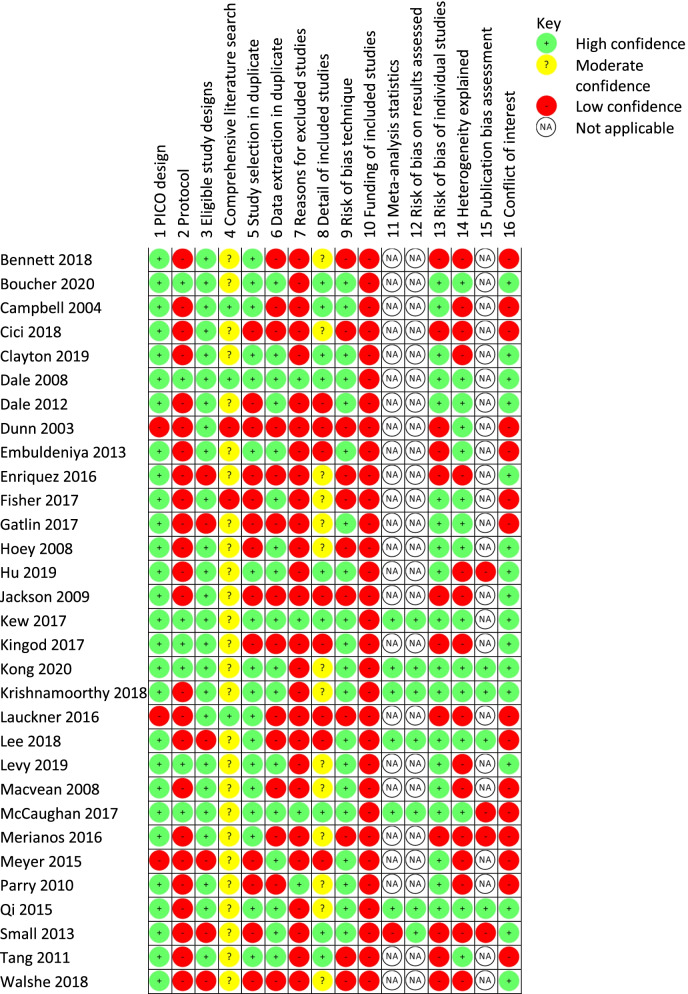
Risk of bias (AMSTAR II)

#### Best evidence synthesis

Due to a lack of ‘high quality’ comprehensive systematic reviews, this best evidence synthesis presents a descriptive summary of Dale and colleagues (2008) and Kew and colleagues (2017) only [46, 53]. Dale and colleagues reviewed across chronic conditions, though included some primary research involving peers without direct experience of a shared chronic condition (i.e., people with shared spiritual beliefs). We abstracted only data concerning people with direct experience of a shared chronic condition (post myocardial infarction or with angina). Peer support included educational telephone-based interventions based in Australia and USA. Dale and colleagues measured health status, mental health, quality of life, self-efficacy, and behaviour change (i.e., diet). No differences were found between any physical health outcomes, mental health, quality of life or self-efficacy. Peer support telephone calls were associated with dietary change in one primary research study only.

Kew and colleagues (2017) reviewed peer support for asthma [53]. Peer support comprised education, strategic thinking, discussion, and encouragement in the context of the Triple A programme that teaches older participants to educate and empower their peers based in Australia, Jordan, and USA. Kew and colleagues measured quality of life, severity of asthma (via exacerbations requiring a course of oral steroids, and asthma control), health care use, health behaviours (i.e., smoking), and adverse events. The asthma-related quality of life random-effects model was imprecise and showed no differences (MD 0.40, 95% confidence interval − 0.02 to 0.81). Most other outcomes did not show an effect favouring peer support. One study found a reduction in adherence in peer support and comparator. Asthma control and nicotine dependence favoured peer support, though this finding was not statistically significant.

Meta-analysis was prevented by heterogeneity between studies, weaknesses in blinding and incomplete reporting. Both reviews shared the conclusion that their findings should be interpreted with caution due to weaknesses in the underlying primary research literature, and that more high-quality clinical trials are indicated.

## Discussion

This systematic review of reviews indicates that peer support may be effective for people with chronic conditions. However, there are methodological weaknesses across the underlying research literature and there is a lack of consistent statistically significant effects of peer support across the included reviews. Therefore, at present it is not possible to draw definitive conclusions about effectiveness, although the potential for peer support interventions is apparent. An obvious difficulty in drawing conclusions is the wide range of intervention components, modalities, and definitions of what constitutes a ‘peer’ identified in this review. Peer support is a complex intervention. Several theories and mechanisms of effect were described in a minority of the reviews included in this study. Peer support was broadly conceptualised as a social intervention. According to *Social identity theory****,*** group membership confers a sense of belonging that acts as a behavioural guide via a set of ingroup social norms [68]. Therefore, it is plausible that the success of peer support may partly depend on how group membership is defined by the recipient of peer support – people with shared clinical characteristics, or people from the same neighbourhood with similar demographics characteristics. Similarly, *social comparison theory* posits that people use peer support to evaluate themselves in relation to others to validate their thoughts, emotions, and experiences, and make positive upward social comparisons to create a sense of hope in a process of recovery or self-management [69]. Understanding how such comparisons might diverge between peers matched on the basis of experiential knowledge of a condition and those with shared demographic factors or other circumstances could help to plan peer support programmes that mitigate the negative impact of less positive social comparisons. Embuldeniya and colleagues (2013) [10] review of mechanisms of peer support reinforces the importance of a shared condition contributing to the bonds formed between people in peer support, and helping others enabled peers to find meaning in their own chronic condition, suggesting that peer support may result in different outcomes depending on how ‘peerness’ is defined. However, relatively few reviews and underpinning primary research explore how peer support works, nor answer specific questions about variation in effectiveness of different types of peers. As a consequence, the extent to which peer support can be optimized by targeting mechanisms of effect remains unclear. Mediation analysis of peer-matching in peer support would be one approach to investigate the importance of this variable, which would inform process measurement in clinical trials.

No single outcome domain was measured across all primary research, suggesting a lack of consensus on what is essential to measure in peer support interventions. This study highlighted that it is common practice to measure clinical recovery in outcome measurement (symptom remission to ‘get back to normal’) alongside *psychosocial processes* of personal recovery such as a sense of connectedness. However, some reviews solely focused on physical symptoms of chronic conditions [55, 63]. Most reviews of peer support synthesised clinical surrogates and mental health outcomes, acknowledging the co-occurrence of chronic physical conditions and mental health problems [70]. However, mental health problems are not simply a symptom of an underlying chronic health condition that can uniformly be addressed by taking away the chronic condition. If peer support does indeed work through creating a sense of connectedness and hope derived from upward social comparisons, it is important to evaluate these processes of personal recovery more consistently in future research [71]. As few as four studies measured a sense of hope and three measured connectedness. The inconsistency in outcome measurement highlighted by this study points toward a lack of consensus regarding what and how to measure when evaluating peer support interventions to enable comparisons in future evidence syntheses which may in turn inform commissioning of new peer support services. A core outcome set is a standardised minimum set of outcomes that should be measured in all clinical trials in an area of health or social care. Research to develop a core outcome set through Delphi consensus methodology would help the research community to bridge this gap.

Confidence in the quality of the included reviews was mostly low or critically low when measured against AMSTAR 2. Most included reviews omitted explicit reference to a protocol established prior to the conduct of the review and a reference to a list of excluded studies with justification for exclusions. Furthermore, many of the included reviews did not assess risk of bias nor robustly conduct study selection and data abstraction in duplicate. Though the authors of the included reviews may not have set out to produce methodologically robust systematic reviews, some of the published reports made conclusive remarks about the effectiveness of peer support without critical appraisal of the primary research informing this view. Therefore, renewed efforts to review the peer support literature ought to be preceded by accessible protocols detailing a robust systematic approach to the literature to facilitate the commissioners’ decision-making process in funding peer support services.

This study presents peer support intervention components summarised in other reviews rather than lengthier descriptions of interventions in primary research. As a result, these data do not represent a complete and detailed catalogue of all published forms of peer support. Furthermore, the use of vote counting in this review of reviews does not provide a robust assessment of the magnitude of effect conferred by intervention components, nor does it account for the relative sizes of the studies included. Due to the variation in outcomes measured, and inconsistent findings between them, it is not possible to conclude what works, for whom, and in what circumstances with these data. The value of understanding the mechanisms of effect in peer support has also been acknowledged in Gopalan and colleagues’ (2017) scoping review of youth peer support for mental health problems [72]. Realist research would be well-suited to exploring these uncertainties to produce a programme theory that explores the mechanisms of effect and highlights the active ingredients to facilitate change in a way that accommodates rather than controls for the heterogeneity across peer support interventions and populations they service.

The evidence base for peer support comprises a wide range of definitions, theories, and methods of peer support. Peers were variably defined as people sharing the same chronic condition, family and friends, community health workers and others in the community, some combination of the above, or not defined at all. This leads to two challenges. Firstly, by attempting to synthesise primary research that adopts different definitions of ‘peerness’ and different theories and components of peer support interventions, evidence syntheses often struggle to compare apples and oranges. Secondly, attempts to pool data from heterogeneous interventions risks overlooking opportunities for learning between different paradigms of peer support across settings and conditions.

This systematic review of reviews focused on peer support between people with a shared chronic condition. In doing so, this review compares a set of interventions that cohere around concepts of shared identity informed by direct lived experience of a chronic condition. However, this prevented any exploration of similarities and differences between different conceptualisations of ‘peerness’. As other gaps in the literature recommended in this discussion are addressed, an update to this review using a meta-narrative design would help to unpick such a heterogeneous topic by comparing and contrasting the different ways in which clinical services and academics have developed and evaluated peer support for people across a broad spectrum of chronic conditions.

## Conclusions

This review presents a range of components of peer support interventions that may be of interest to clinicians when developing new peer support programmes. However, there is a lack of high-quality primary research (outside of studies on cancer, cardiovascular disease, and somatic illness) underpinning few robust systematic reviews, which limits understanding of what components of peer support to use and with whom. Until this changes, the implementation of peer support in different clinical settings may benefit from participatory action research so that services may reflect patient preference at a local level. Furthermore, for those clinical services intending to implement novel peer support interventions, this review provides a matrix of mechanisms of effect that may guide theory-driven intervention development.

Though four major chronic conditions (cancer, diabetes, cardiovascular, and chronic respiratory disease) are well-represented in reviews focusing exclusively on peer support between people with a chronic condition, there was a marked absence of reviews on many other prevalent chronic conditions with different pathologies and lived experience. This review highlights a window of opportunity to scope the literature to determine whether there is a need for further primary research or systematic reviews of peer support for chronic conditions absent from this review.

## Supplementary Information

**Additional file 1.**

**Additional file 2.**

**Additional file 3.**

**Additional file 4.**

**Additional file 5.**

## Data Availability

Included study data are included in this report’s supplementary information files.

## Abbreviations

AMSTAR: A Measurement Tool to Assess Systematic Reviews
CDSMP: Chronic Disease Self-Management Program
EPP: Expert Patient Programme
MD: Mean difference
PICOS: Population, Intervention, Comparison, Outcome, and Study design
PROSPERO: International Prospective Register of Systematic Reviews
WHO: World Health Organization
