# Supplementary material for: Peer support for people with chronic conditions: a systematic review of reviews
Source: BMC Health Serv Res. 2022 Mar 31;22:427. doi: 10.1186/s12913-022-07816-7 (PMC8973527; doi:10.1186/s12913-022-07816-7)
Supplement: Supplementary file 2 — Additional file 2. [file 12913_2022_7816_MOESM2_ESM.docx]

**Additional file 2** .docx; Search terms

| Ovid MEDLINE(R) 1946 to September Week 4 2019 |
| --- |
| 1. (peer* adj2 (support* or specialist* or service* or employ* or work* or provide* or trainer* or run or ran or organi* or managed or led or directed or delivered or operated or assist* or companion*)).tw,kw. 2. ((consumer*) adj2 (support* or specialist* or service* or employ* or work* or provide* or trainer* or run or ran or organi* or managed or led or directed or delivered or operated or assist* or companion*)).tw,kw. 3. exp Chronic Disease/ 4. (chronic condition* or chronic disease* or chronic illness*).tw,kw. 5. exp HIV Infections/ OR Cerebral Palsy/ OR exp Spinal Dysraphism/ OR exp Neoplasms/ OR exp Diabetes Mellitus/ OR exp Dementia/ OR exp Parkinsonian Disorders/ or Parkinson Disease/ OR exp Epilepsy/ OR Multiple Sclerosis/ OR Migraine Disorders* OR exp Vision Disorders/ OR exp Hearing disorders/ OR exp Cardiovascular Diseases/ OR exp Respiratory Tract Diseases/ OR exp Musculoskeletal Diseases/ OR exp Pain/ OR exp Burns/ OR Chronic Encephalopathy/ OR Chronic Brain Injury/ OR exp Liver cirrhosis/ OR exp Hepatitis/ 6. (HIV OR AIDS OR Human Immunodeficiency Virus OR Acquired Immunodeficiency Syndrome OR Acute Retroviral Syndrome OR Cerebral Palsy OR Spina bifida OR Spinal Dysraphism OR Neoplas* OR Cancer OR Tumor OR Carcino* or Onco* OR Diabet* OR Prediabet* OR Dementia OR Alzheimer OR Aphasia OR Creutzfeldt-Jakob OR Huntington OR Lewy Body OR Posterior Cortical Atrophy OR Parkinson* OR Epilep* OR Multiple Sclerosis OR Migraine OR Mild Cognitive Impairment OR Vision disorder* OR Vision loss OR Visual impairment* OR Blind* OR Amblyopia OR Diplopia OR Hemianopsia OR Photophobia OR Scotoma OR Hearing disorder* OR Hearing loss OR Deaf* OR Hyperacusis OR Tinnitus OR Cardiovascular Disease* OR Heart Disease* OR Vascular Disease* OR Stroke OR Brain Infarction OR Circulatory Disease* OR Hypertension OR Respiratory Tract Disease* OR Respiratory Disease* OR Asthma OR Chronic Obstructive Pulmonary Disease OR COPD OR Bronchi* OR Pulmonary OR Cystic Fibrosis OR Lung disease* OR Thoracic OR Tracheal OR Musculoskeletal disease* OR Arthritis OR Osteoarthritis OR Gout OR Fibromyalgia OR Rheumat* OR Pain OR Burn* OR Encephalopathy OR Brain injury OR Cirrhosis OR Hepatitis).tw,kw. 7. Systematic review/ or Review/ 8. (review).tw,kw. 9. 1 or 2 10. 3 or 4 or 5 or 6 11. 7 or 8 12. 9 and 10 and 11 |
