# Supplementary material for: Peer support for people with chronic conditions: a systematic review of reviews
Source: BMC Health Serv Res. 2022 Mar 31;22:427. doi: 10.1186/s12913-022-07816-7 (PMC8973527; doi:10.1186/s12913-022-07816-7)
Supplement: Supplementary file 3 — Additional file 3. [file 12913_2022_7816_MOESM3_ESM.docx]

**Additional file 3** .docx; List of excluded publications and reasons for exclusion

| **Publication** | **Reason for exclusion** |
| --- | --- |
| Ado Rivera, I. B. A systematic review of economic evaluations of peer support interventions for adults with type II diabetes mellitus. | Insufficient data |
| Afshar R, Tang TS, Askari AS, et al. (2020). Peer support interventions in type 2 diabetes: Review of components and process outcomes. Journal of Diabetes, 12(4):315-338. DOI: 10.1111/1753-0407.12999 | Data inseparable |
| Alderson, G. H. K. (2007). An investigation of gay men's experiences with HIV counselling and peer support services. Canadian Journal of Community Mental Health. Retrieved from http://www.cjcmh.com/doi/abs/10.7870/cjcmh-2007-0017 | Article inaccessible |
| Andersson, G. L., Brjann; Weise, Cornelia. (2011). Internet-delivered treatment to promote health. Current opinion in psychiatry, 24(2), 168-172. doi:https://dx.doi.org/10.1097/YCO.0b013e3283438028 | Peer support not the primary focus of review |
| Badger, K. A., Amy; Peterson, Pam. (2017). Aftercare, Survivorship, and Peer Support. Clinics in plastic surgery, 44(4), 885-891. doi:https://dx.doi.org/10.1016/j.cps.2017.05.020 | Ineligible intervention |
| Bain-Brickley, D. B., Lisa M.; Kennedy, Gail E.; Rutherford, George W. (2011). Interventions to improve adherence to antiretroviral therapy in children with HIV infection. The Cochrane database of systematic reviews(12), CD009513. doi:https://dx.doi.org/10.1002/14651858.CD009513 | Insufficient data |
| Bal, M. I. S., Jane N. T.; Roelofs, Pepijn D. D. M.; Bal, Roland; van Staa, AnneLoes; Miedema, Harald S. (2016). Exploring effectiveness and effective components of self-management interventions for young people with chronic physical conditions: A systematic review. Patient education and counseling, 99(8), 1293-1309. doi:https://dx.doi.org/10.1016/j.pec.2016.02.012 | Data inseparable |
| Barbosa, H. C. O., J. A. Q.; Costa, J. M.; Santos, R. P.; Goncalves Miranda, L.; Ribeiro, D. D.; Dusse, L.; Torres, H. C.; Martins, M. A. P. (2019). Evaluation of strategies focused on empowerment and change of behavior with a potential utility in patients with thromboembolic disorders. Research and Practice in Thrombosis and Haemostasis, 3(Supplement 1), 829-830. doi:http://dx.doi.org/10.1002/rth2.12229 | Data inseparable |
| Barone, M. T. U. V., M. A.; Madden, P. B. (2016). Are diabetes camps effective? Diabetes Research and Clinical Practice, 114, 15-22. doi:http://dx.doi.org/10.1016/j.diabres.2016.01.013 | Ineligible population |
| Bell, B. F. L. (2015). Improving the transition to adult care for young people with chronic kidney disease: Springer. | Peer support not the primary focus of review |
| Bender, J. L. R., Arun; Diorio, Caroline; Englesakis, Marina; Jadad, Alejandro R. (2011). Can pain be managed through the Internet? A systematic review of randomized controlled trials. Pain, 152(8), 1740-1750. doi:https://dx.doi.org/10.1016/j.pain.2011.02.012 | Peer support not the primary focus of review |
| Bloomfield, G. S. V., Rajesh; Vasudevan, Lavanya; Kithei, Anne; Were, Martin; Velazquez, Eric J. (2014). Mobile health for non-communicable diseases in Sub-Saharan Africa: a systematic review of the literature and strategic framework for research. Globalization and health, 10, 49. doi:https://dx.doi.org/10.1186/1744-8603-10-49 | Ineligible intervention |
| Bradford, N. K. C., Raymond Javan. (2017). Health promotion and psychological interventions for adolescent and young adult cancer survivors: A systematic literature review. Cancer treatment reviews, 55, 57-70. doi:https://dx.doi.org/10.1016/j.ctrv.2017.02.011 | Peer support not the primary focus of review |
| Breakey, V. R., Bouskill, V., Nguyen, C., Luca, S., Stinson, J. N., & Ahola Kohut, S. (2018). Online Peer-to-Peer Mentoring Support for Youth with Hemophilia: Qualitative Needs Assessment. *JMIR pediatrics and parenting*, *1*(2), e10958. https://doi.org/10.2196/10958 | Ineligible methods |
| Brett Thombs, V. D. S. G. G. E.-B. V. M. R. R. M. S. Examining the effectiveness of support groups for people with medical illness: a systematic review. | Insufficient data |
| Brown, R. J. P., G.; Bollard, M.; Thake, D.; McGregor, G. (2019). Very brief interventions for risk reducing behaviour in type 2 diabetes: A scoping review. Diabetic Medicine, 36(Supplement 1), 97-98. doi:http://dx.doi.org/10.1111/dme.13883 | Article inaccessible |
| Brownson, C. A. H., M. (2009). The role of peer support in diabetes care and self-management. Patient, 2(1), 5-17. doi:http://dx.doi.org/10.2165/01312067-200902010-00002 | Data inseparable |
| Burton, J., Eggleston, B., Brenner, J., Truchil, A., Zulkiewicz, B. A., & Lewis, M. A. (2017). Community-Based Health Education Programs Designed to Improve Clinical Measures Are Unlikely to Reduce Short-Term Costs or Utilization Without Additional Features Targeting These Outcomes. *Population health management*, *20*(2), 93–98. https://doi.org/10.1089/pop.2015.0185 | Ineligible methods |
| Butow, P. U., J.; Kirsten, L.; Wain, G.; Hobbs, K.; Zordan, R.; Sandoval, M.; Smith, K.; Stenlake, A.; Price, M.; Juraskova, I. (2009). Cancer support group research: An example of collaborative work in Australia. Psycho-oncology, 18(SUPPL. 2), S30. doi:http://dx.doi.org/10.1002/pon.1594 | Ineligible methods |
| Campbell, H. S. P., Marie Rose; Deane, Karen. (2004). Cancer peer support programs-do they work? Patient education and counseling, 55(1), 3-15. Retrieved from http://ovidsp.ovid.com/ovidweb.cgi?T=JS&PAGE=reference&D=med5&NEWS=N&AN=15476984 | Duplicate |
| Carpenter, R. D., Toni; Barker, Kendra. (2019). Interventions for self-management of type 2 diabetes: An integrative review. International journal of nursing sciences, 6(1), 70-91. doi:https://dx.doi.org/10.1016/j.ijnss.2018.12.002 | Peer support not the primary focus of review |
| Cherrington, A. M., Michelle Y.; Hayes, Michaela; Halanych, Jewell H.; Wright, Mary Annette; Appel, Susan J.; Andreae, Susan J.; Safford, Monika. (2012). Intervention mapping as a guide for the development of a diabetes peer support intervention in rural Alabama. Preventing chronic disease, 9, E36. Retrieved from http://ovidsp.ovid.com/ovidweb.cgi?T=JS&PAGE=reference&D=med8&NEWS=N&AN=22239751 | Ineligible methods |
| Chung, G. M. R. (2013). Systematic review of positive youth development programs for adolescents with chronic illness. Pediatrics. Retrieved from https://pediatrics.aappublications.org/content/131/5/e1605.abstract | Peer support not the primary focus of review |
| Clark, M. (2008). Diabetes self-management education: a review of published studies. Primary care diabetes, 2(3), 113-120. doi:https://dx.doi.org/10.1016/j.pcd.2008.04.004 | Ineligible intervention |
| Clark, Ella V; (2018) The feasibility of a novel group self-management intervention for stroke. Doctoral thesis (Ph.D), UCL (University College London) | Ineligible peers |
| Clark, E. MacCrosain, A. Ward, NS. Jones, F. (2020). The key features and role of peer support within group self-management interventions for stroke? A systematic review. Disability and Rehabilitation, 42(3) pp. 307-316. doi:[10.1080/09638288.2018.1498544](https://doi.org/10.1080/09638288.2018.1498544) | Ineligible peers |
| Clemente, D., Leon, L., Foster, H., Minden, K., & Carmona, L. (2016). Systematic review and critical appraisal of transitional care programmes in rheumatology. *Seminars in arthritis and rheumatism*, *46*(3), 372–379. https://doi.org/10.1016/j.semarthrit.2016.06.003 | Peer support not the primary focus of review |
| Coffey, L. M., Orla; Dunne, Simon; Sharp, Linda; Timmons, Aileen; Desmond, Deirdre; O'Sullivan, Eleanor; Timon, Conrad; Gooberman-Hill, Rachael; Gallagher, Pamela. (2016). Cancer survivors' perspectives on adjustment-focused self-management interventions: a qualitative meta-synthesis. Journal of cancer survivorship : research and practice, 10(6), 1012-1034. Retrieved from http://ovidsp.ovid.com/ovidweb.cgi?T=JS&PAGE=reference&D=med12&NEWS=N&AN=27150211 | Ineligible intervention |
| Colella, T. J. F. K., Kathryn M. (2004). Peer support. An under-recognized resource in cardiac recovery. European journal of cardiovascular nursing : journal of the Working Group on Cardiovascular Nursing of the European Society of Cardiology, 3(3), 211-217. Retrieved from http://ovidsp.ovid.com/ovidweb.cgi?T=JS&PAGE=reference&D=med5&NEWS=N&AN=15350230 | Ineligible intervention |
| Connelly, J. M., J.; Kirk, A.; MacRury, S. (2013). Impact of interventional components on the effectiveness of technology-based physical activity promotion in Type 2 diabetes management: A systematic review. Diabetic Medicine, 30(SUPPL. 1), 124-125. doi:http://dx.doi.org/10.1111/dme.12091_2 | Ineligible intervention |
| Cotter, A. P. D., Nefertiti; Agne, April A.; Cherrington, Andrea L. (2014). Internet interventions to support lifestyle modification for diabetes management: a systematic review of the evidence. Journal of diabetes and its complications, 28(2), 243-251. doi:https://dx.doi.org/10.1016/j.jdiacomp.2013.07.003 | Peer support not the primary focus of review |
| CRAIG, A. The Role of Peer Support in Facilitating Psychosocial Adjustment to Chronic Illness in. | Ineligible methods |
| Crane-Okada, R., Freeman, E., Kiger, H., Ross, M., Elashoff, D., Deacon, L., & Giuliano, A. E. (2012). Senior peer counseling by telephone for psychosocial support after breast cancer surgery: effects at six months. *Oncology nursing forum*, *39*(1), 78–89. https://doi.org/10.1188/12.ONF.78-89 | Ineligible methods |
| Crawford, S., & Bath, N. (2013). Peer support models for people with a history of injecting drug use undertaking assessment and treatment for hepatitis C virus infection. *Clinical infectious diseases : an official publication of the Infectious Diseases Society of America*, *57 Suppl 2*, S75–S79. https://doi.org/10.1093/cid/cit297 | Ineligible peers |
| de Bruin, S. R., Versnel, N., Lemmens, L. C., Molema, C. C., Schellevis, F. G., Nijpels, G., & Baan, C. A. (2012). Comprehensive care programs for patients with multiple chronic conditions: a systematic literature review. *Health policy (Amsterdam, Netherlands)*, *107*(2-3), 108–145. https://doi.org/10.1016/j.healthpol.2012.06.006 | Data inseparable |
| Delisle, V. C. G., Stephanie T.; Kloda, Lorie A.; Boruff, Jill; El-Baalbaki, Ghassan; Korner, Annett; Malcarne, Vanessa L.; Thombs, Brett D.; Scleroderma Support Group Project Advisory, Team. (2016). Effect of support group peer facilitator training programmes on peer facilitator and support group member outcomes: a systematic review. BMJ open, 6(11), e013325. doi:https://dx.doi.org/10.1136/bmjopen-2016-013325 | Insufficient data |
| Doctor, M. E. (1992). Helping the burned child to adapt. Clinics in plastic surgery, 19(3), 607-614. Retrieved from http://ovidsp.ovid.com/ovidweb.cgi?T=JS&PAGE=reference&D=med3&NEWS=N&AN=1633668 | Article inaccessible |
| Dressler, C. (2013). Improving the uptake of cardiac rehabilitation in invited patients: a multi-method evaluation. University of York (United Kingdom), Retrieved from http://search.ebscohost.com/login.aspx?direct=true&db=jlh&AN=109865390&site=ehost-live Available from EBSCOhost jlh database. (Ph.D.) | Peer support not the primary focus of review |
| Dwarswaard, J. B., E. J. M.; van Staa, A.; Boeije, H. R. (2016). Self-management support from the perspective of patients with a chronic condition: a thematic synthesis of qualitative studies. Health Expectations, 19(2), 194-208. doi:10.1111/hex.12346 | Peer support not the primary focus of review |
| Emeka Okonji, Z. O. F. M. S. V. B. V. W. Psychosocial support interventions for improved adherence and retention to ART care for adolescents and young people living with HIV: a systematic review with narrative analysis. | Insufficient data |
| Evans, L. B., C. (2008). The efficacy of community-based rehabilitation programmes for adults with TBI...including commentary by Thomas Y. International Journal of Therapy & Rehabilitation, 15(10), 446-458. Retrieved from http://search.ebscohost.com/login.aspx?direct=true&db=jlh&AN=105698764&site=ehost-live | Article inaccessible |
| Fisher, E. B. C., Muchieh Maggy; Parada, Humberto; Robinette, Jennifer B.; Tang, Patrick Y.; Urlaub, Diana M.; Castillo, Claudia; Guzman-Corrales, Laura M.; Hino, Sayaka; Hunter, Jaimie; Katz, Ariana W.; Symes, Yael R.; Worley, Heidi P.; Xu, Cuirong. (2014). Peer support in health care and prevention: cultural, organizational, and dissemination issues. *Annual review of public health, 35*, 363-383. doi:https://dx.doi.org/10.1146/annurev-publhealth-032013-182450 | Data inseparable |
| Foster, G., Taylor, S. J., Eldridge, S. E., Ramsay, J., & Griffiths, C. J. (2007). Self-management education programmes by lay leaders for people with chronic conditions. *The Cochrane database of systematic reviews*, (4), CD005108. https://doi.org/10.1002/14651858.CD005108.pub2 | Ineligible intervention |
| Foster, D. (2016). Defining a model of cancer survivorship from a psychosocial perspective: Increasing patient well-being during survivorship. Journal of Psychosocial Oncology, 34(1-2), 105-106. doi:http://dx.doi.org/10.1080/07347332.2016.1147913 | Peer support not the primary focus of review |
| Frazier, S. K. D. D. T. (2008). Meta-analysis of psychological interventions to promote adherence to treatment in pediatric chronic health conditions. Journal of Pediatric Psychology. Retrieved from https://academic.oup.com/jpepsy/article-abstract/33/6/590/1746406 | Data inseparable |
| Funnell, M. (2009). Peer-based behavioural strategies to improve chronic disease self-management and clinical outcomes: evidence, logistics, evaluation considerations and needs for …. Family practice. Retrieved from https://academic.oup.com/fampra/article-abstract/27/suppl_1/i17/640066 | Data inseparable |
| Furler, I. B. C. W. M. M. J. Evaluating Peer Support for People with Chronic Conditions. | Insufficient data |
| Galdas, P. F., Jennifer; Bower, Peter; Kidd, Lisa; Blickem, Christian; McPherson, Kerri; Hunt, Kate; Gilbody, Simon; Richardson, Gerry. (2015). The effectiveness of self-management support interventions for men with long-term conditions: a systematic review and meta-analysis. BMJ open, 5(3), e006620. doi:https://dx.doi.org/10.1136/bmjopen-2014-006620 | Peer support not the primary focus of review |
| Ghahramani, N. (2015). Potential impact of peer mentoring on treatment choice in patients with chronic kidney disease: a review. Archives of Iranian Medicine (AIM). Retrieved from http://search.ebscohost.com/login.aspx?direct=true&profile=ehost&scope=site&authtype=crawler&jrnl=10292977&AN=101656192&h=X4TiC%2FRoH7vyW%2FmQ3u%2BIvNZPMxsxD%2BCnrnhM6Mu%2BiXnR%2B2IgOOUuvjIi%2BbTRNarkDayLFfb7eibh%2B9CxWrDqcg%3D%3D&crl=c | Data inseparable |
| Ginis, K. A. M. N., C. R.; Smith, A. L. (2013). Peer-delivered physical activity interventions: An overlooked opportunity for physical activity promotion. Translational behavioral medicine, 3(4), 434-443. doi:http://dx.doi.org/10.1007/s13142-013-0215-2 | Ineligible intervention |
| Gleeson, H. P., S. (2015). What are the best practices in effective, high-quality HIV support for women in the UK? HIV Medicine, 16(SUPPL. 2), 72. doi:http://dx.doi.org/10.1111/hiv.12265 | Ineligible methods |
| Gobble, A. (2018). Empowerment-based nutrition education series in a peer support group setting for type 2 diabetes. Journal of Investigative Medicine, 66(1), 186. doi:http://dx.doi.org/10.1136/jim-2017-000663.288 | Ineligible intervention |
| Govindasamy, D. K., K.; Ford, N. (2014). Strengthening the HIV cascade to ensure an effective future ART response in sub-Saharan Africa. Transactions of the Royal Society of Tropical Medicine and Hygiene, 108(1), 1-+. doi:10.1093/trstmh/trt105 | Ineligible intervention |
| Govindasamy, D. M., J.; Negussi, E. K.; Baggaley, R. C.; Ford, N.; Kranzer, K. (2014). Interventions to improve or facilitate linkage to or retention in pre-ART (HIV) care and initiation of ART in low- and middle-income settings - a systematic review. Journal of the International AIDS Society, 17, 24. doi:10.7448/ias.17.1.19032 | Ineligible intervention |
| Gucciardi, E. J.-P., N.; Karam, G.; Sidani, S. (2016). Designing and delivering facilitated storytelling interventions for chronic disease self-management: a scoping review. BMC health services research, 16, 249. doi:http://dx.doi.org/10.1186/s12913-016-1474-7 | Insufficient data |
| Haines, K. J. B., S. J.; Hopkins, R. O.; McPeake, J.; Quasim, T.; Ritchie, K.; Iwashyna, T. J. (2018). Peer support in critical care: A systematic review. Critical care medicine, 46(9), 1522-1531. doi:http://dx.doi.org/10.1097/CCM.0000000000003293 | Ineligible intervention |
| Harris J, Springett J, Croot L, Booth A, Campbell F, Thompson J, et al. (2015) Can community-based peer support promote health literacy and reduce inequalities? A realist review. Public Health Res, 3(3) | Peer support not the primary focus of review |
| Heisler, M., Halasyamani, L., Cowen, M. E., Davis, M. D., Resnicow, K., Strawderman, R. L., Choi, H., Mase, R., & Piette, J. D. (2013). Randomized controlled effectiveness trial of reciprocal peer support in heart failure. *Circulation. Heart failure*, *6*(2), 246–253. https://doi.org/10.1161/CIRCHEARTFAILURE.112.000147 | Ineligible methods |
| Hogan, B. E. L., W.; Najarian, B. (2002). Social support - Do they interventions work? Clinical psychology review, 22(3), 381-440. doi:10.1016/s0272-7358(01)00102-7 | Insufficient data |
| Hogan, B. E. L., Wolfgang; Najarian, Bahman. (2002). Social support interventions: do they work? Clinical psychology review, 22(3), 383-442. Retrieved from http://ovidsp.ovid.com/ovidweb.cgi?T=JS&PAGE=reference&D=med4&NEWS=N&AN=17201192 | Data inseparable |
| Hogan, B. E. L., Wolfgang; Najarian, Bahman. (2002). Social support interventions: do they work? Clinical psychology review, 22(3), 383-442. Retrieved from http://ovidsp.ovid.com/ovidweb.cgi?T=JS&PAGE=reference&D=med4&NEWS=N&AN=17201192 | Duplicate |
| Hughes, R., Fleming, P., & Henshall, L. (2020). Peer support groups after acquired brain injury: a systematic review. *Brain injury*, *34*(7), 847–856. https://doi.org/10.1080/02699052.2020.1762002 | Ineligible population |
| Humphreys, K. R., J. (1994). RESEARCHING SELF-HELP MUTUAL AID GROUPS AND ORGANIZATIONS - MANY ROADS, ONE JOURNEY. Applied & Preventive Psychology, 3(4), 217-231. doi:10.1016/s0962-1849(05)80096-4 | Article inaccessible |
| Huntley, A. M., T.; King, A.; Evans, M.; Persad, R.; Sharp, D. (2015). Supportive care interventions for men with prostate cancer: A systematic review. Supportive Care in Cancer, 23(1 SUPPL. 1), S369. doi:http://dx.doi.org/10.1007/s00520-015-2712-y | Ineligible intervention |
| Ihrig, A., Karschuck, P., Haun, M. W., Thomas, C., & Huber, J. (2020). Online peer-to-peer support for persons affected by prostate cancer: A systematic review. *Patient education and counseling*, *103*(10), 2107–2115. https://doi.org/10.1016/j.pec.2020.05.009 | Ineligible population |
| Ivery, J. D. (2018). Evidence-Based Strategies and Practices to Manage Veterans' Noncancer Pain: A Systematic Review. (D.N.P.). Walden University, Ann Arbor. Retrieved from https://search.proquest.com/docview/2021741405?accountid=8630; https://birmingham-primo.hosted.exlibrisgroup.com/openurl/44BIR/44BIR_Services?genre=dissertations+%26+theses&issn=&title=Evidence-Based+Strategies+and+Practices+to+Manage+Veterans%27+Noncancer+Pain%3A+A+Systematic+Review&volume=&issue=&date=2018&atitle=&spage=&sid=ProQuest+Dissertations+%26+Theses+Global&author=Ivery ProQuest Dissertations & Theses Global database. (10747632) | Insufficient data |
| Jones, S. (2016). Experiences of Healthcare Professionals and Patients in Paediatric Cystic Fibrosis: Making and Breaking Bonds. (Ph.D.). Bangor University (United Kingdom), Ann Arbor. Retrieved from https://search.proquest.com/docview/1885889535?accountid=8630; https://birmingham-primo.hosted.exlibrisgroup.com/openurl/44BIR/44BIR_Services?genre=dissertations+%26+theses&issn=&title=Experiences+of+Healthcare+Professionals+and+Patients+in+Paediatric+Cystic+Fibrosis%3A+Making+and+Breaking+Bonds&volume=&issue=&date=2016&atitle=&spage=&sid=ProQuest+Dissertations+%26+Theses+Global&author=Jones; http://e.bangor.ac.uk/9762/ ProQuest Dissertations & Theses Global database. (10589894) | Ineligible intervention |
| Juma, K. R., Michael; Roy, Monika; Vorkoper, Susan; Temu, Tecla M.; Levitt, Naomi S.; Oladepo, Oladimeji; Zakus, David; Yonga, Gerald. (2018). From HIV prevention to non-communicable disease health promotion efforts in sub-Saharan Africa: A Narrative Review. AIDS (London, England), 32 Suppl 1, S63-S73. doi:https://dx.doi.org/10.1097/QAD.0000000000001879 | Ineligible intervention |
| Kanters, S. P., Jay Jh; Chan, Keith; Ford, Nathan; Forrest, Jamie; Thorlund, Kristian; Nachega, Jean B.; Mills, Edward J. (2016). Use of peers to improve adherence to antiretroviral therapy: a global network meta-analysis. Journal of the International AIDS Society, 19(1), 21141. doi:https://dx.doi.org/10.7448/IAS.19.1.21141 | Ineligible intervention |
| Kanters, S. P., J. H.; Chan, K.; Ford, N.; Forrest, J.; Thorlund, K.; Nachega, J. B.; Mills, E. J. (2016). Use of peers to improve adherence to antiretroviral therapy: A global network meta-analysis: A. Journal of the International AIDS Society, 19(1), 21141. doi:http://dx.doi.org/10.7448/IAS.19.1.21141 | Duplicate |
| Kew, K. M. C., Robin; Crossingham, Iain. (2017). Lay-led and peer support interventions for adolescents with asthma. *The Cochrane database of systematic reviews, 4*, CD012331. doi:https://dx.doi.org/10.1002/14651858.CD012331.pub2 | Duplicate |
| Kimberley Haines, S. B. R. H. J. M. T. Q. K. R. T. J. I. Peer support to improve patient and family outcomes following critical illness: a systematic review. | Ineligible intervention |
| Kloseck, S. A. K. K. J. M. M. (2018). Education and social support as key factors in osteoarthritis management programs: a scoping review. Arthritis. Retrieved from https://www.hindawi.com/journals/arthritis/2018/2496190/abs/ | Data inseparable |
| Kobra, R. M., Safavi; Hossain, Yahyavi Seyed; Hojatollah, Farahani. (2017). Peer-Based Interventions Focused on Face to Face and Telephone Call Models in Type II Diabetes Management. Journal of Diabetic Nursing, 5(2), 123-138. Retrieved from http://search.ebscohost.com/login.aspx?direct=true&db=jlh&AN=124459957&site=ehost-live | Article inaccessible |
| Kong, L.-N. H., Ping; Yang, Li; Cui, Dan. (2019). The effectiveness of peer support on self-efficacy and quality of life in adults with type 2 diabetes: A systematic review and meta-analysis. Journal of advanced nursing, 75(4), 711-722. doi:https://dx.doi.org/10.1111/jan.13870 | Ineligible peers |
| Kong, L.-N. H., Ping; Yang, Li; Cui, Dan. (2019). The effectiveness of peer support on self-efficacy and quality of life in adults with type 2 diabetes: A systematic review and meta-analysis. Journal of advanced nursing, 75(4), 711-722. doi:https://dx.doi.org/10.1111/jan.13870 | Duplicate |
| Kornhaber, R., Visentin, D., Kaji Thapa, D., West, S., Haik, J., & Cleary, M. (2020). Burn camps for burns survivors-Realising the benefits for early adjustment: A systematic review. *Burns: journal of the International Society for Burn Injuries*, *46*(1), 33–43. https://doi.org/10.1016/j.burns.2018.12.005 | Ineligible peers |
| Kowitt, S. D., Ellis, K. R., Carlisle, V., Bhushan, N. L., Black, K. Z., Brodar, K., Cranley, N. M., Davis, K. L., Eng, E., Martin, M. Y., McGuirt, J., Sokol, R. L., Tang, P. Y., Vines, A. I., Walker, J. S., & Fisher, E. B. (2019). Peer support opportunities across the cancer care continuum: a systematic scoping review of recent peer-reviewed literature. *Supportive care in cancer : official journal of the Multinational Association of Supportive Care in Cancer*, *27*(1), 97–108. https://doi.org/10.1007/s00520-018-4479-4 | Ineligible peers |
| Lafond, N. A. C., R.; Pandya, H.; Smyth, A.; Williams, J.; Elliott, R. A. (2010). Medicine-taking interventions in children and young people: One size fits all, one size fits none. *International Journal of Pharmacy Practice, 18*(SUPPL. 1), 11-12. doi:http://dx.doi.org/10.1111/j.2042-7174.2010.00010.x | Data inseparable |
| Lake, A. J. H., K.; Jones, C.; Johnston, S.; Taverna, G.; Speight, J. (2019). What do we know about health professional-convened diabetes peer support? a scoping review of the literature. Diabetic Medicine, 36(Supplement 1), 110. doi:http://dx.doi.org/10.1111/dme.13883 | Article inaccessible |
| Lambert, S. M. D., Joseph; Bodiroza, Aleksandar; Martin, Jack; Staunton, Shaun; Walker, Rebecca. (2013). Effective peer education in HIV: defining factors that maximise success. Sexual Health (14485028), 10(4), 325-331. doi:10.1071/SH12195 | Ineligible methods |
| Lawn, S. S., Adrian. (2010). Supporting self-management of chronic health conditions: common approaches. *Patient education and counseling, 80*(2), 205-211. doi:https://dx.doi.org/10.1016/j.pec.2009.10.006 | Peer support not the primary focus of review |
| Leonard, K. Q., Alexandria C.; Lindsay, J. Michael. (2015). Moderated Social Media Support Groups for Patients. *Journal of Consumer Health on the Internet, 19*(3/4), 219-232. doi:10.1080/15398285.2015.1089397 | Ineligible intervention |
| Ling-Na, K. The effects of peer support on diabetes distress and depression in adults with type 2 diabetes: a systematic review and meta-analysis. | Insufficient data |
| Linnan, L. F., Edwin B.; Hood, Sula. (2013). The power and potential of peer support in workplace interventions. American Journal of Health Promotion, 28(1), TAHP-2-TAHP-10. Retrieved from http://search.ebscohost.com/login.aspx?direct=true&db=jlh&AN=107915429&site=ehost-live | Data inseparable |
| Locher, C. M., M.; Gaab, J.; Gerger, H. (2019). Long-Term Effects of Psychological Interventions to Improve Adherence to Antiretroviral Treatment in HIV-Infected Persons: A Systematic Review and Meta-Analysis. AIDS patient care and STDs, 33(3), 131-144. doi:10.1089/apc.2018.0164 | Article inaccessible |
| Lorthios-Guilledroit, A. R., L.; Filiatrault, J. (2018). Factors associated with the implementation of community-based peer-led health promotion programs: A scoping review. Evaluation and program planning, 68, 19-33. doi:10.1016/j.evalprogplan.2018.01.008 | Data inseparable |
| Maclachlan LR, Mills K, Lawford B, Egerton T, Setchell J, Hall LM, Plinsinga M, Besomi M, Teo S, Eyles JP, Mellor R, Hodges PW, Hunter DJ, Vicenzino B, Bennell KL  Design, effectiveness and implementation of peer-to-peer online support groups for people with chronic musculoskeletal disorders: a systematic review  JMIR Preprints. 09/08/2019:15822 DOI: 10.2196/preprints.15822 | Insufficient data |
| MacPherson, P. M., Chigomezgo; Ferguson, Jane; Armstrong, Alice; Kranzer, Katharina; Ferrand, Rashida A.; Ross, David A. (2015). Service delivery interventions to improve adolescents' linkage, retention and adherence to antiretroviral therapy and HIV care. Tropical medicine & international health : TM & IH, 20(8), 1015-1032. doi:https://dx.doi.org/10.1111/tmi.12517 | Ineligible intervention |
| MacRury, J. C. A. K. J. M. S. (2013). The use of technology to promote physical activity in Type 2 diabetes management: a systematic review. Diabetic Medicine. Retrieved from https://onlinelibrary.wiley.com/doi/abs/10.1111/dme.12289 | Ineligible peers |
| Malcomson, K. S. (2007). Psychosocial well-being and quality of life in individuals with Multiple Sclerosis: the effect of psychosocial interventions. (Ph.D.). University of Ulster (United Kingdom), Ann Arbor. Retrieved from https://search.proquest.com/docview/301675952?accountid=8630; https://birmingham-primo.hosted.exlibrisgroup.com/openurl/44BIR/44BIR_Services?genre=dissertations+%26+theses&issn=&title=Psychosocial+well-being+and+quality+of+life+in+individuals+with+Multiple+Sclerosis%3A+the+effect+of+psychosocial+interventions&volume=&issue=&date=2007&atitle=&spage=&sid=ProQuest+Dissertations+%26+Theses+Global&author=Malcomson ProQuest Dissertations & Theses Global database. (U221672) | Article inaccessible |
| Malcomson, K. S. D., L.; Lowe-Strong, A. S. (2007). Psychosocial interventions in people with multiple sclerosis - A review. Journal of Neurology, 254(1), 1-13. doi:10.1007/s00415-006-0349-y | Ineligible intervention |
| Maria Sammut, C. E. N. F. H. J. Systematic review of strategies and interventions which aim to increase time spent in moderate to vigorous physical activity (MVPA) following a transient ischemic attack (TIA) or non-disabling stroke<br />. | Insufficient data |
| Mariano, T. Y. W., L. M.; Edwards, R. R.; Jamison, R. N. Online teletherapy for chronic pain: A systematic review. Journal of telemedicine and telecare, 14. doi:10.1177/1357633x19871746 | Article inaccessible |
| Martini, J. T.-T., A.; Dujardin, B.; Macq, J.; Gobatto, I. (2011). Patient involvement in care strategies for chronic diseases: the cases of diabetes and HIV/AIDS care in Mali. What lessons can be learned? *Tropical Medicine and International Health, 16*(SUPPL. 1), 326. Retrieved from http://ovidsp.ovid.com/ovidweb.cgi?T=JS&PAGE=reference&D=emed12&NEWS=N&AN=70589870 | Data inseparable |
| Mashiko, N. K., Y.; Kobayashi, M.; Tsumura, A.; Fujimaki, I.; Ishigaki, M.; Toju, K.; Wakiguchi, Y.; Ohta, R. (2018). Review of the literature on peer support of adolescent and young adult cancer patients. *Pediatric Blood and Cancer, 65*(Supplement 2), S619. doi:http://dx.doi.org/10.1002/pbc.27455 | Insufficient data |
| Matthias, M. S., Kukla, M., McGuire, A. B., Damush, T. M., Gill, N., & Bair, M. J. (2016). Facilitators and Barriers to Participation in a Peer Support Intervention for Veterans With Chronic Pain. *The Clinical journal of pain*, *32*(6), 534–540. https://doi.org/10.1097/AJP.0000000000000297 | Ineligible methods |
| Mawson, C. A. E. (2019). Peer Support for Chronic and Complex Conditions: chronicillness.org.au. | Ineligible peers |
| McCann, L. M., Kathryn Anne; Pugh, Gemma. (2019). Digital Interventions to Support Adolescents and Young Adults With Cancer: Systematic Review. JMIR cancer, 5(2), e12071. doi:https://dx.doi.org/10.2196/12071 | Ineligible intervention |
| McElfish, P. A. P., Rachel S.; Esquivel, Monica K.; Sinclair, Ka'imi A.; Townsend, Claire; Hawley, Nicola L.; Haggard-Duff, Lauren K.; Kaholokula, Joseph Keawe'aimoku. (2019). Diabetes Disparities and Promising Interventions to Address Diabetes in Native Hawaiian and Pacific Islander Populations. Current diabetes reports, 19(5), N.PAG-N.PAG. doi:10.1007/s11892-019-1138-1 | Data inseparable |
| McEvoy, C. M., E.; McKinley, M.; Woodside, J. (2015). A systematic review of the effectiveness of peer support to reduce cardiovascular risk. Annals of Nutrition and Metabolism, 67(SUPPL. 1), 316. doi:http://dx.doi.org/10.1159/000440895 | Data inseparable |
| McEvoy, C. T., McAuley, E., Moore, S. E., Cupples, M., Kee, F., Young, I. S., … Woodside, J. V. (2017). A systematic review and meta-analysis of the effectiveness of peer support to reduce cardiovascular risk. *Proceedings of the Nutrition Society*, *76*(OCE3), E117. http://doi.org/10.1017/S0029665117001902 | Ineligible peers |
| McGrath, C. S., Kiranpreet; Mahl, Harpreet. (2017). Interventions that facilitate the occupational engagement of older adults with age-related vision loss: Findings from a scoping review. Physical & Occupational Therapy in Geriatrics, 35(1), 3-19. doi:http://dx.doi.org/10.1080/02703181.2016.1267292 | Peer support not the primary focus of review |
| McKellar, J. M., D.; Mackie, J. (2012). Peers fostering hope. Stroke, 43(11), e138. doi:http://dx.doi.org/10.1161/01.str.0000422054.44193.87 | Ineligible methods |
| Melissa Oxlad, E. D. Peer support programs for people with cancer: a systematic review. | Insufficient data |
| Metcalfe, D. R. D., A. J.; Olufajo, O. A.; Massa, M. S.; Ketelaar, Nabm; Flottorp, S. A.; Perry, D. C. (2018). Impact of public release of performance data on the behaviour of healthcare consumers and providers. Cochrane Database of Systematic Reviews(9). doi:10.1002/14651858.CD004538.pub3 | Ineligible intervention |
| Meyer, P. (2004). Consumer representation in multi-site HIV, mental health, and substance abuse research: The HIV/AIDS Treatment Adherence, Health Outcomes and Cost Study. *AIDS Care - Psychological and Socio-Medical Aspects of AIDS/HIV, 16*(SUPPL. 1), S137-S153. doi:http://dx.doi.org/10.1080/09540120412331315240 | Data inseparable |
| Mills, E. J. L., R.; Thorlund, K.; Lorenzi, M.; Muldoon, K.; Kanters, S.; Linnemayr, S.; Gross, R.; Calderon, Y.; Amico, K. R.; Thirumurthy, H.; Pearson, C.; Remien, R. H.; Mbuagbaw, L.; Thabane, L.; Chung, M. H.; Wilson, I. B.; Liu, A.; Uthman, O. A.; Simoni, J.; Bangsberg, D.; Yaya, S.; Barnighausen, T.; Ford, N.; Nachega, J. B. (2014). Interventions to promote adherence to antiretroviral therapy in Africa: a network meta-analysis. The Lancet HIV, 1(3), e104-e111. doi:http://dx.doi.org/10.1016/S2352-3018%2814%2900003-4 | Data inseparable |
| Mills, K. A. M., L. R.; Lawford, B.; Besomi, M.; Egerton, T.; Eyles, J.; Hall, L. M.; Melo, L.; Mellor, R.; Plisinga, M. L.; Robbins, S. R.; Setchell, J.; Hunter, D. J.; Vicenzino, B.; Bennell, K. L. (2019). The design, user characteristics and efficacy of online support groups for arthritis and other chronic musculoskeletal disorders: a systematic review. Osteoarthritis and Cartilage, 27(Supplement 1), S451. doi:http://dx.doi.org/10.1016/j.joca.2019.02.488 | Ineligible peers |
| Mirzoshoev, H. W., S. (2016). Managing diabetes and co-morbid mental health problems in low and middle income countries: A scoping literature review. Annals of Global Health, 82(3), 445. Retrieved from http://ovidsp.ovid.com/ovidweb.cgi?T=JS&PAGE=reference&D=emed17&NEWS=N&AN=614045003 | Data inseparable |
| Moore, S. E., McEvoy, C. T., McKinley, M. C., & Woodside, J. V. (2017). The effectiveness of peer support in encouraging dietary behaviour change in adults: a systematic review. *Proceedings of the Nutrition Society*, *76*(OCE3), [E111]. https://doi.org/10.1017/S0029665117001847 | Ineligible peers |
| Morello, E. M. S. L. C. O. A. (2019). Self-management programs for Aboriginal and Torres Strait Islander Peoples with chronic conditions: A rapid review. Chronic illness. Retrieved from https://journals.sagepub.com/doi/abs/10.1177/1742395317750266 | Peer support not the primary focus of review |
| Mueller, C. W., S.; Nestmann, F.; Stubbs, B.; Bebbington, P.; Raymont, V. (2018). Interventions to enhance coping after traumatic brain injury: A systematic review. *International Journal of Therapy and Rehabilitation, 25*(3), 107-119. doi:10.12968/ijtr.2018.25.3.107 | Ineligible peers |
| Narcisse, L. W., Edward A.; Hsu, Lewis L. (2018). Summer Camps for Children with Sickle Cell Disease. The Ochsner journal, 18(4), 358-363. doi:https://dx.doi.org/10.31486/toj.18.0045 | Ineligible peers |
| Newby, T. A. G., Julie N.; Ganzini, Linda K.; McDonagh, Marian S. (2015). Interventions that may reduce depressive symptoms among prostate cancer patients: a systematic review and meta-analysis. Psycho-oncology, 24(12), 1686-1693. doi:https://dx.doi.org/10.1002/pon.3781 | Peer support not the primary focus of review |
| O'Brien, N. H., Quan Nha; Law, Susan; Massoud, Sarah; Carter, Allison; Kaida, Angela; Loutfy, Mona; Cox, Joseph; Andersson, Neil; de Pokomandy, Alexandra. (2018). Health System Features That Enhance Access to Comprehensive Primary Care for Women Living with HIV in High-Income Settings: A Systematic Mixed Studies Review. AIDS patient care and STDs, 32(4), 129-148. doi:https://dx.doi.org/10.1089/apc.2017.0305 | Article inaccessible |
| O'Hara, M. C. H., L.; O'Donnell, M.; Nery, N.; Byrne, M.; Heller, S. R.; Dinneen, S. F.; Irish Type 1 Diabetes Young Adult Study, Group. (2017). A systematic review of interventions to improve outcomes for young adults with Type 1 diabetes. Diabetic medicine : a journal of the British Diabetic Association, 34(6), 753-769. doi:https://dx.doi.org/10.1111/dme.13276 | Peer support not the primary focus of review |
| Oates, E. W. L. E. T. F. J. (2017). Effective self-management interventions for patients with lupus: potential impact of peer mentoring. The American journal of the …. Retrieved from https://www.sciencedirect.com/science/article/pii/S0002962917300137 | Ineligible intervention |
| Odgers-Jewell, K. B., L. E.; Kelly, J. T.; Isenring, E. A.; Reidlinger, D. P.; Thomas, R. (2017). Effectiveness of group-based self-management education for individuals with Type 2 diabetes: a systematic review with meta-analyses and meta-regression. Diabetic medicine : a journal of the British Diabetic Association, 34(8), 1027-1039. doi:https://dx.doi.org/10.1111/dme.13340 | Peer support not the primary focus of review |
| ÖZer, N. G. V., Fatma. (2018). Yanıklı Çocukların Ailelerinde Destek Grup Girişimi Yapılmalı mıdır? Turkiye Klinikleri Hemsirelik Bilimleri, 10(2), 153-159. doi:10.5336/nurses.2017-57835 | Article not in English language |
| Parvin, L. S., S.; Maharaj, N.; Miller, A.; Kazanjian, A. (2015). Addressing psychosocial needs of canadian men with prostate cancer and their partners: Development of a peer navigation support program. Psycho-oncology, 24(SUPPL. 2), 182-183. doi:http://dx.doi.org/10.1002/pon.3874 | Data inseparable |
| Patil, S. J. R., Todd; Koopman, Richelle J.; Lindbloom, Erik J.; Elliott, Susan G.; Mehr, David R.; Conn, Vicki S. (2016). Peer Support Interventions for Adults With Diabetes: A Meta-Analysis of Hemoglobin A1c Outcomes. *Annals of family medicine, 14*(6), 540-551. doi:https://dx.doi.org/10.1370/afm.1982 | Ineligible peers |
| Patil, S. J. R., Todd; Koopman, Richelle J.; Lindbloom, Erik J.; Elliott, Susan G.; Mehr, David R.; Conn, Vicki S. (2018). Effect of peer support interventions on cardiovascular disease risk factors in adults with diabetes: a systematic review and meta-analysis. BMC public health, 18(1), 398. doi:https://dx.doi.org/10.1186/s12889-018-5326-8 | Ineligible peers |
| Payne, J. A. (1995). Group learning for adults with disabilities or chronic disease. Rehabilitation nursing : the official journal of the Association of Rehabilitation Nurses, 20(5), 268-272. Retrieved from http://ovidsp.ovid.com/ovidweb.cgi?T=JS&PAGE=reference&D=med3&NEWS=N&AN=7569305 | Ineligible intervention |
| Pedersen, A. H., Thomas F. (2010). Pilots of oncology health care: A concept analysis of the patient navigator role. Oncology nursing forum, 37(1), 55-60. doi:http://dx.doi.org/10.1188/10.ONF.55-60 | Peer support not the primary focus of review |
| Pennington, M. V., Shelina; Donaldson, Cam; White, Martin; Lhussier, Monique; Deane, Katherine; Forster, Natalie; Carr, Susan M. (2013). Cost-effectiveness of health-related lifestyle advice delivered by peer or lay advisors: synthesis of evidence from a systematic review. Cost effectiveness and resource allocation : C/E, 11(1), 30. doi:https://dx.doi.org/10.1186/1478-7547-11-30 | Data inseparable |
| Perlman, L. M., Cohen, J. L., Altiere, M. J., Brennan, J. A., Brown, S. R., Mainka, J. B., & Diroff, C. R. (2010). A multidimensional wellness group therapy program for veterans with comorbid psychiatric and medical conditions. Professional Psychology: Research and Practice, 41(2), 120–127. https://doi.org/10.1037/a0018800 | Ineligible methods |
| Peter MacPherson, C. M. D. R. Service delivery interventions to improve adolescents’ retention and adherence to antiretroviral therapy in adolescents to HIV care: a systematic review. | Ineligible intervention |
| Philippou, K. L., E.; Ktisti, S.; Kyriakou, M.; Middleton, N. (2016). Social support and adherence to the therapy in patients with heart failure: A systematic review. European Journal of Heart Failure, 18(SUPPL. 1), 401. doi:http://dx.doi.org/10.1002/ejhf.539 | Data inseparable |
| Pike, A. R. (2014). *The parts are greater than the sum of the whole: exploring the process of change in a pain management programme using single case study design.* (Ph.D.). University of London, University College London (United Kingdom), Ann Arbor. | Ineligible intervention |
| Plow, M. A. F., M.; Rezac, M. (2011). A Scoping Review of Self-Management Interventions for Adults With Multiple Sclerosis. *Pm&R, 3*(3), 251-262. doi:10.1016/j.pmrj.2010.11.011 | Insufficient data |
| Pomery, A. S., Penelope; Xhilaga, Miranda; Gough, Karla. (2016). Skills, knowledge and attributes of support group leaders: A systematic review. Patient education and counseling, 99(5), 672-688. doi:https://dx.doi.org/10.1016/j.pec.2015.11.017 | Ineligible population |
| Price, N. K., S. (2009). How Effective is Peer Education in Addressing Young People's Sexual and Reproductive Health Needs in Developing Countries? Children & Society, 23(4), 291-302. doi:10.1111/j.1099-0860.2008.00175.x | Ineligible population |
| Ramadas, A. Q., K. F.; Chan, C. K. Y.; Oldenburg, B. (2011). Web-based interventions for the management of type 2 diabetes mellitus: a systematic review of recent evidence. *International journal of medical informatics, 80*(6), 389-405. doi:https://dx.doi.org/10.1016/j.ijmedinf.2011.02.002 | Insufficient data |
| Ramchand, R., Ahluwalia, S. C., Xenakis, L., Apaydin, E., Raaen, L., & Grimm, G. (2017). A systematic review of peer-supported interventions for health promotion and disease prevention. *Preventive medicine*, *101*, 156–170. https://doi.org/10.1016/j.ypmed.2017.06.008 | Ineligible population |
| Reichmann, J. P. B., K. R. (2018). An integrative review of peer support for patients undergoing major limb amputation. Journal of Vascular Nursing, 36(1), 34-39. doi:10.1016/j.jvn.2017.10.002 | Ineligible population |
| Rice, D. B. T., B. D. (2019). Support Groups in Scleroderma. Current Rheumatology Reports, 21(4), 7. doi:10.1007/s11926-019-0808-y | Ineligible methods |
| Roe, C. T., Cathrine; Howe, Emilie Isager; Tenovuo, Olli; Azouvi, Philippe; Andelic, Nada. (2019). Randomized Controlled Trials of Rehabilitation Services in the Post-acute Phase of Moderate and Severe Traumatic Brain Injury - A Systematic Review. Frontiers in neurology, 10, 557. doi:https://dx.doi.org/10.3389/fneur.2019.00557 | Data inseparable |
| Rose-Clarke, K. B., Abigail; Marston, Cicely; Prost, Audrey. (2019). Peer-facilitated community-based interventions for adolescent health in low- and middle-income countries: A systematic review. PloS one, 14(1), e0210468. doi:https://dx.doi.org/10.1371/journal.pone.0210468 | Ineligible intervention |
| Rowshanak Afshar, T. T. A. A. H. B. D. S. A systematic review of recruitment, training, and health-related outcomes for individuals who deliver peer support in type 2 diabetes. | Peer support not the primary focus of review |
| Ruddock, J. S. P., M.; Gary-Webb, T. L.; Walker, E. A.; Davis, N. J. (2016). Innovative strategies to improve diabetes outcomes in disadvantaged populations. Diabetic Medicine, 33(6), 723-733. doi:10.1111/dme.13088 | Data inseparable |
| Rutherford, C. Z., N. (2017). Strategies to support shared decision making in breast cancer. Cancer Forum, 41(1). Retrieved from http://cancerforum.org.au/wp-content/uploads/2017/02/2.-Strategies-to-support-decision-making-in-breast-cancer-Rutherford_CR.pdf; http://ovidsp.ovid.com/ovidweb.cgi?T=JS&PAGE=reference&D=emed18&NEWS=N&AN=617500909 | Article inaccessible |
| Saboo, B. K., S.; Raghupathy, P.; Bandgar, T.; Chawdhary, S.; Vishwanathan, V.; Chugh, S. (2018). Care triad for diabetes in children & adolescents-diabetes education counseling & support. *Pediatric Diabetes, 19*(Supplement 26), 39. doi:http://dx.doi.org/10.1111/pedi.12745 | Data inseparable |
| Sadler, E. S., S.; Tinker, A.; Bhalla, A.; McKevitt, C. (2017). Developing a novel peer support intervention to promote resilience after stroke. *Health & Social Care in the Community, 25*(5), 1590-1600. doi:10.1111/hsc.12336 | Data inseparable |
| Salander, P. (2010). Facilitating interventions and/or relationships in malignant brain tumors. Advances in therapy, 27(1), 17-27. doi:https://dx.doi.org/10.1007/s12325-010-0003-z | Data inseparable |
| Santiago de Araújo Pio, C. C., G. S. S.; Davies, P.; Taylor, R. S.; Grace, S. L. (2019). Interventions to promote patient utilisation of cardiac rehabilitation. Cochrane Database of Systematic Reviews(2). doi:10.1002/14651858.CD007131.pub4 | Peer support not the primary focus of review |
| Sazlina, S.-G. B., Colette; Yasin, Shajahan. (2013). Interventions to promote physical activity in older people with type 2 diabetes mellitus: a systematic review. Frontiers in public health, 1, 71. doi:https://dx.doi.org/10.3389/fpubh.2013.00071 | Peer support not the primary focus of review |
| Scanlon, M. L. V., Rachel C. (2013). Current strategies for improving access and adherence to antiretroviral therapies in resource-limited settings. HIV/AIDS (Auckland, N.Z.), 5, 1-17. doi:https://dx.doi.org/10.2147/HIV.S28912 | Ineligible intervention |
| Schubart, J. R., Stuckey, H. L., Ganeshamoorthy, A., & Sciamanna, C. N. (2011). Chronic health conditions and internet behavioral interventions: a review of factors to enhance user engagement. *Computers, informatics, nursing : CIN*, *29*(2), 81–92. https://doi.org/10.1097/NCN.0b013e3182065eed | Peer support not the primary focus of review |
| Shapiro, K. R., Sunanda. (2007). Sexual health for people living with HIV. *Reproductive health matters, 15*(29 Suppl), 67-92. Retrieved from http://ovidsp.ovid.com/ovidweb.cgi?T=JS&PAGE=reference&D=med5&NEWS=N&AN=17531749 | Data inseparable |
| Siegel, K. R. Z., X.; Peng Ng, B.; Jawanda, S.; Proia, K.; Zhang, X.; Zhang, P. (2018). Cost-effectiveness of interventions to prevent and manage diabetes-a systematic review and update. *Diabetes, 67*(Supplement 1), A334. Retrieved from http://diabetes.diabetesjournals.org/content/suppl/2018/07/05/67.Supplement_1.DC1; http://ovidsp.ovid.com/ovidweb.cgi?T=JS&PAGE=reference&D=emexa&NEWS=N&AN=623565771 | Data inseparable |
| Simmons, D. V., Ja; Rush, Elaine; Dear, Murray. (2010). The New Zealand experience in peer support interventions among people with diabetes. Family practice, 27 Suppl 1, i53-61. doi:https://dx.doi.org/10.1093/fampra/cmp012 | Data inseparable |
| Simoni, J. M. N., Kimberly M.; Franks, Julie C.; Yard, Samantha S.; Lehavot, Keren. (2011). Are peer interventions for HIV efficacious? A systematic review. *AIDS and behavior, 15*(8), 1589-1595. doi:https://dx.doi.org/10.1007/s10461-011-9963-5 | Insufficient data |
| Smith, R. (2015). Investigating the impact of volunteer mentoring on carers of people with dementia and volunteer mentors. (Ph.D.). Kingston University (United Kingdom), Ann Arbor. Retrieved from https://search.proquest.com/docview/1837040047?accountid=8630; https://birmingham-primo.hosted.exlibrisgroup.com/openurl/44BIR/44BIR_Services?genre=dissertations+%26+theses&issn=&title=Investigating+the+impact+of+volunteer+mentoring+on+carers+of+people+with+dementia+and+volunteer+mentors&volume=&issue=&date=2015&atitle=&spage=&sid=ProQuest+Dissertations+%26+Theses+Global&author=Smith ProQuest Dissertations & Theses Global database. (10293019) | Ineligible intervention |
| South, J. B., Anne-Marie; Woodall, James. (2017). Developing a Typology for Peer Education and Peer Support Delivered by Prisoners. Journal of Correctional Health Care, 23(2), 214-229. doi:10.1177/1078345817700602 | Ineligible population |
| Steele, R. L., A. (2013). Telehealth and ubiquitous computing for bandwidth-constrained rural and remote areas. Personal and Ubiquitous Computing, 17(3), 533-543. doi:10.1007/s00779-012-0506-5 | Data inseparable |
| Stein, K. P., B.; Dunsiger, S. (2015). The role of peer support for physical well-being after cancer. Psycho-oncology, 24(SUPPL. 2), 2. doi:http://dx.doi.org/10.1002/pon.3873 | Ineligible methods |
| Stinson, J. N. K., S. A.; Amaria, K.; Bell, M. J.; Forgeron, P.; Kaufman, M.; Luca, N.; Spiegel, L. R. (2015). Virtual peer-to-peer mentoring support for adolescents with juvenile idiopathic arthritis: The virtual peer-to-peer program. Arthritis and Rheumatology, 67(SUPPL. 10). doi:http://dx.doi.org/10.1002/art.39448 | Ineligible methods |
| Stubbs, B. W., Julie; Shannon, Jennifer; Gaughran, Fiona; Craig, Tom. (2016). Peer support interventions seeking to improve physical health and lifestyle behaviours among people with serious mental illness: A systematic review. International journal of mental health nursing, 25(6), 484-495. doi:https://dx.doi.org/10.1111/inm.12256 | Ineligible population |
| Tolley, J. S., & Foroushani, P. S. (2014). What do we know about one-to-one peer support for adults with a burn injury? A scoping review. *Journal of burn care & research : official publication of the American Burn Association*, *35*(3), 233–242. https://doi.org/10.1097/BCR.0b013e3182957749 | Ineligible peers |
| Turner, K. A. R., Danielle B.; Carboni-Jimenez, Andrea; Boruff, Jill; Thombs, Brett D. (2019). Effects of training and support programs for leaders of illness-based support groups: commentary and updated evidence. Systematic reviews, 8(1), 67. doi:https://dx.doi.org/10.1186/s13643-019-0981-0 | Data inseparable |
| van Dam, H. A. v. d. H., Frans G.; Knoops, Lut; Ryckman, Richard M.; Crebolder, Harry F. J. M.; van den Borne, Bart H. W. (2005). Social support in diabetes: a systematic review of controlled intervention studies. Patient education and counseling, 59(1), 1-12. Retrieved from http://ovidsp.ovid.com/ovidweb.cgi?T=JS&PAGE=reference&D=med5&NEWS=N&AN=16198213 | Peer support not the primary focus of review |
| Vanden Berg, K. C., P.; Cail, A.; Brady, M. (2015). Peer-support for people with aphasia following stroke: A systematic review. International Journal of Stroke, 10(SUPPL. 5), 17. doi:http://dx.doi.org/10.1111/ijs.12634-3 | Insufficient data |
| Veinot, P. B., M.; Nyhof-Young, J.; Perrier, L.; Sargeant, J.; Tugwell, P.; Reeves, S.; Sale, J. (2009). Peer to peer mentoring: Facilitating individuals with early inflammatory arthritis to manage their arthritis. Journal of Rheumatology, 36(11), 2588-2589. doi:http://dx.doi.org/10.3899/jrheum.090763 | Insufficient data |
| Vickie Rowland, L. H. M. C. C. M. The effectiveness of psychosocial interventions on the psychological well-being of stroke survivors in the sub-acute stage of stroke: a systematic review. | Insufficient data |
| Villarreal-Garza, C. M.-C., B. A.; Platas, A.; Ramos-Elias, P. (2015). Specialized programs to support young women with breast cancer. Current opinion in supportive and palliative care, 9(3), 308-316. doi:10.1097/spc.0000000000000155 | Article inaccessible |
| Visser-Meily, A. v. H., Caroline; Post, Marcel; Schepers, Vera; Lindeman, Eline. (2005). Intervention studies for caregivers of stroke survivors: a critical review. Patient education and counseling, 56(3), 257-267. Retrieved from http://ovidsp.ovid.com/ovidweb.cgi?T=JS&PAGE=reference&D=med5&NEWS=N&AN=15721967 | Ineligible population |
| Warner, G. P., Tanya; Villeneuve, Michelle; Audulv, Asa; Versnel, Joan. (2015). A systematic review of the effectiveness of stroke self-management programs for improving function and participation outcomes: self-management programs for stroke survivors. Disability and rehabilitation, 37(23), 2141-2163. doi:https://dx.doi.org/10.3109/09638288.2014.996674 | Peer support not the primary focus of review |
| Warren, J. (2006). IT-empowered consumers and advocates for chronic disease management. Healthcare Review Online, 10(3). Retrieved from https://www.scopus.com/inward/record.uri?eid=2-s2.0-33749007157&partnerID=40&md5=b316bc4f583aea56da8705b708c57d28 | Data inseparable |
| Weller, C. D. B., Rachelle; Johnston, Renea V. (2016). Interventions for helping people adhere to compression treatments for venous leg ulceration. The Cochrane database of systematic reviews, 3, CD008378. doi:https://dx.doi.org/10.1002/14651858.CD008378.pub3 | Ineligible intervention |
| Werfalli, M. R., Peter; Engel, Mark; Peer, Nasheeta; Kalula, Sebastiana; Kengne, Andre P.; Levitt, Naomi S. (2015). Effectiveness of community-based peer-led diabetes self-management programmes (COMP-DSMP) for improving clinical outcomes and quality of life of adults with diabetes in primary care settings in low and middle-income countries (LMIC): a systematic review and meta-analysis. BMJ open, 5(7), e007635. doi:https://dx.doi.org/10.1136/bmjopen-2015-007635 | Insufficient data |
| Wheeler, S. A.-V., Amanda; Davis, Diana. (2016). Effectiveness of Interventions to Improve Occupational Performance for People With Psychosocial, Behavioral, and Emotional Impairments After Brain Injury: A Systematic Review. The American journal of occupational therapy : official publication of the American Occupational Therapy Association, 70(3), 7003180060p7003180061-7003180069. doi:https://dx.doi.org/10.5014/ajot.115.020677 | Article inaccessible |
| Wilcock, K. C. S. (2013). The effectiveness of peer support interventions for community dwelling adults with chronic non-cancer pain: a systematic review. International Journal of Evidence-Based …. Retrieved from https://journals.lww.com/ijebh/Fulltext/2013/09000/The_effectiveness_of_peer_support_interventions.16.aspx | Article inaccessible |
| Williams, D. A. (2012). Review of internet-based interventions for pain finds some evidence to support the effectiveness of cognitive and behavioural interventions, but further quality study is needed to assess the effect of peer support and social networking programmes. Evidence-based nursing, 15(2), 41-42. doi:https://dx.doi.org/10.1136/ebnurs.2011.100293 | Ineligible methods |
| Williams, H. T., Paula. (2016). Sickle Cell Disease: A Review of Nonpharmacological Approaches for Pain. Journal of pain and symptom management, 51(2), 163-177. doi:https://dx.doi.org/10.1016/j.jpainsymman.2015.10.017 | Ineligible intervention |
| Wilson, H. C. J. H. P. (2012). Self-management for people with long-term neurological conditions. British journal of community …. Retrieved from https://www.magonlinelibrary.com/doi/abs/10.12968/bjcn.2012.17.6.250 | Ineligible intervention |
| Wobma, R. N., Rinske H. M.; Ket, Johannes C. F.; Kwakkel, Gert. (2016). Evidence for peer support in rehabilitation for individuals with acquired brain injury: A systematic review. Journal of rehabilitation medicine, 48(10), 837-840. doi:https://dx.doi.org/10.2340/16501977-2160 | Insufficient data |
| Woolley, E. (2012). Literature Review: Assisting Older People with Chronic Conditions through Self-Management Support: pdfs.semanticscholar.org. | Ineligible intervention |
| Wu, Y. C. Y., Y. S.; Kornelius, E.; Lu, Y. L.; Li, C. J.; Lin, Y. T.; Chen, Y. J.; Huang, C. N. (2014). Comparison of self-monitoring blood glucose integrated into a peer-support group with usual care education in type 2 diabetes. Diabetes, 63(SUPPL. 1), A173. doi:http://dx.doi.org/10.2337/db14-665-832 | Ineligible intervention |
| Xue, L. (2014). *Effectiveness of a peer leader supported diabetes self-management support program on patient assessment of care for chronic conditions (PACIC)*: d-scholarship.pitt.edu. | Peer support not the primary focus of review |
| Zebrack, B. I., Sinead. (2012). Psychosocial care of adolescent and young adult patients with cancer and survivors. Journal of clinical oncology : official journal of the American Society of Clinical Oncology, 30(11), 1221-1226. doi:https://dx.doi.org/10.1200/JCO.2011.39.5467 | Peer support not the primary focus of review |
| Zhang, X. Y., Shuaishuai; Sun, Kaige; Fisher, Edwin B.; Sun, Xinying. (2016). How to achieve better effect of peer support among adults with type 2 diabetes: A meta-analysis of randomized clinical trials. Patient education and counseling, 99(2), 186-197. doi:https://dx.doi.org/10.1016/j.pec.2015.09.006 | Data inseparable |
| Zhong, C. S. M.-T., G. J. (2017). The effect of peer-led self-management education programmes for adolescents with asthma: A systematic review and meta-analysis. Health Education Journal, 76(6), 676-694. doi:http://dx.doi.org/10.1177/0017896917712297 | Data inseparable |
| 杨雪柯, 王., ; 杨建国,; 张梦霞,; 郭晓贝,. (2019). 同伴支持对乳腺癌病人生命质量影响的 系统评价. Chinese Nursing Research, 33(15), 2573-2577. doi:10.12102/j.issn.1009-6493.2019.15.005 | Article not in English language |
