# Supplementary material for: Peer support for people with chronic conditions: a systematic review of reviews
Source: BMC Health Serv Res. 2022 Mar 31;22:427. doi: 10.1186/s12913-022-07816-7 (PMC8973527; doi:10.1186/s12913-022-07816-7)
Supplement: Supplementary file 4 — Additional file 4. [file 12913_2022_7816_MOESM4_ESM.docx]

**Additional file 4** .docx; Study datasets

| **Study ID** | **Review type** | **Review aim** | **Primary research study design** | **Setting** | **Country** | **Recipient demographics** |
| --- | --- | --- | --- | --- | --- | --- |
| Acquired brain injury, cerebral palsy, and spina bifida | | | | | | |
| Levy 2019 | Systematic review | To determine the impact of peer support interventions for adults with acquired brain injury, cerebral palsy, and spina bifida on community integration and QoL; and to identify the key characteristics of peer support interventions for adults with acquired brain injury, cerebral palsy, and spina bifida. | RCT only | 1. Multiple rehabilitation centres (2) 2. Multiple hospitals (1) 3. Rehabilitation hospital (1) 4. Stroke rehabilitation centre (1) 5. Multiple rehabilitation hospitals (1) | 1. Netherlands (2) 2. USA (2) Australia (1) UK (1) | - Age range (average):  31.7 – 69.4  - Percentage female range:  11 – 59 |
| Asthma | | | | | | |
| Kew 2017 | Meta-analysis | To find out whether sessions led by peers or by lay leaders (i.e. not healthcare professionals) help to reduce these risks [of poor adherence with treatment] and lead to better asthma control. | 1. CRT (3)  2. RCT (2) | 1. High school (2) 2. Male school (1) 3. Camp (1) | 1. Jordan (2) 2. USA (1) 3. Australia (1) | - Age (range):  12 – 17  - Percentage: male: 60% |
| Cancer | | | | | | |
| Campbell 2004 | Systematic review | What types of cancer peer support programs have been evaluated? What do we know about participants? What benefits, risks and barriers are associated with cancer peer support programs? | 1. Needs assessment (2)  2. Exploratory and purposive interviews (4)  3. Participant observation (1)  4. Focus groups (1)  5. Survey: no comparison group (5) 6. Survey: comparison groups (3) 7. RCT (3) | Not reported | Not reported | - Age (range):  47 – 56  - Percentage female range:  34-87 |
| Dunn 2003 | Literature review | To assess the prevalence and contribution of articles on peer support published in the research literature on psychosocial oncology over the past decade | 1. Descriptive (15)  2. Cross-sectional post-test evaluations (5)  3. Case comparison (2) 4. Experimental study (1)  5. RCT (1) | Not reported | Not reported | 1. Women (11)  2. Men (4) 3. Adolescents (2)  4. Hispanic (1) |
| Hoey 2008 | Systematic review | To identify the different models of peer support described in the literature for people with cancer, and examine the research assessing their effectiveness | 1. One group descriptive data (26) 2. Non-randomized comparative studies (9)  3.  RCT (10) | 1. Home, hospital, public area  2. Hospital/ treatment centre  3. Public area (2)  4. Workplace  5. Hospital (3)  6. Educational institutions, hospitals, medical clinics  7. Home (10)  8. Health-care, educational and community centres; | 1. Canada (6) 2. Sweden (1) 3. Australia (2) 4. USA (23) 5. UK (3) 6. Scandinavia (1) 7. Ireland (1)  8. Netherlands (1) | Not reported |
| Lee 2018 | Meta‐analysis | To determine the effects of a specific model of peer support (one-on-one or face-to-face peer-led supportive care) | 1. RCT (11)  2. Non-randomized comparative studies (2) | Not reported | 1. Canada (2) 2. USA (8) 3. Australia (1) 4. South Korea (2) | - Age range (average):  46 – 60 |
| Macvean 2008 | Systematic review | To conduct a systematic review of literature reporting on the use of volunteers in support programs for people with cancer | 1. One-group descriptive data (6) 2. Non-randomized comparative studies (4) 3. RCT (2) | Not reported | 1. Israel (1) 2. USA (8);  3. Ireland 4. Canada (2) 5. Australia (2) | 1. African American (1) |
| Meyer 2015 | Literature review | What are the objectives and outcomes of studies assessing one-to-one peer support in cancer care; and what is the quality of the programme description (including the description of providers and recipients of one-to-one peer support) and the quality of the research methodology of the reviewed studies? | 1. RCT (4) 2. Non-randomised comparative studies (1) 3. One-group descriptive data (8) | Not specified | 1. USA (6) 2. Australia (3) 3. UK (2) 4. South Korea (1) | 1. Latinas (1) 2. African-American (1) |
| Walshe 2018 | Scoping review | What is the experience and impact of different forms of peer support for people with advanced cancer? | 1. Survey (2)  2. Qualitative (3)  3. RCT (4)  4. Cross sectional comparison study (1) | Not reported | 1. US (7)  2. Australia (1)  3. Denmark (1)  4. Germany (1) | - Age range (average):  49 – 62 |
| Hu 2019 | Systematic review | To determine the effects of different types of peer support interventions and to provide guidance and reference for health workers who want to design and conduct such programs for this patient population in the future | RCT (15) | Not reported | Not reported | 1. African American women (3) 2. Spanish-speaking women (1) 3. Women with propensity for anxiety and depression (1) |
| McCaughan 2017 | Systematic review | To assess effects of online support groups on the emotional distress, uncertainty, anxiety, depression and quality of life (QoL) of women with breast cancer | RCT (3) | Not reported | USA (3) | 1. Mean age was 52.95 (M) and 51.57 (peer-led Ninety per cent were ‘white’ and 10% ‘African American’ 2. Almost 40% of participants were younger than 50 years of age, and 92% were described as ’white’ 3. Mean age of participants was 52.7 years, and all were described as ’white’ |
| Cardiovascular Disease | | | | | | |
| Clayton 2019 | Scoping review | To develop a greater understanding of the current state of evidence of interventions used to modify social support in people with CVD | 1. RCT (4) 2. Pre-post feasibility (2) | Not reported | 1. USA (3) 2. Canada (2) 3. Germany (1) | - Age range (average):  57 – 72.6  - Percentage female range:  51.9 – 100 |
| Jackson 2009 | Literature review | To establish whether self-help groups offer or indicate potential for CHD rehabilitation and secondary prevention. | 1. Cross-sectional study (1)  2. Retrospective interviews (1)  3. Longitudinal study (3) | Community-based (5) | Sweden (5) | Not reported |
| Parry 2010 | Systematic review | To critically examine the effects of peer support interventions on health outcomes for individuals with heart disease. | RCT (5) | Not reported | 1. USA (3)  2. Australia 3. (1) 4. Canada (1) | 1. Aged 15–74 in urban Australia, English speaking (1)  2. Males, mean age 56.5 years, 1st-time elective CABG surgery in urban Quebec (1)  3. All men, mean age 59 years (1) 4. A suburban community in South West USA (1)  5, Unpartnered adults >65 y of age (1) |
| Small 2013 | Meta‐analysis | To assess the evidence on the effectiveness and cost-effectiveness of telephone self-management interventions led by ‘lay health workers’ and ‘peer support workers’ to support the prevention and management of vascular disease. | RCT (6) | 1. Primary care and community setting (4) 2. Secondary care (1) 3. US Department of Veterans Affairs (1) | 1. USA (4) 2. UK (1) 3. Canada (1) | 1. African American women (1) 2. Unpartnered, ≥65 years (1) |
| Chronic disease | | | | | | |
| Embuldeniya 2013 | Qualitative systematic review/qualitative evidence synthesis | To synthesise qualitative literature about the perceived impact and experience of participating in peer support interventions for individuals with chronic disease | Qualitative research (22) | 1. Community settings (1) 2. Clinic (1) | Not reported | Not reported |
| Enriquez 2016 | Systematic review | To explore the literature with regard to peer-facilitated medication adherence interventions and to examine what is known about the effectiveness of such interventions in chronic disease | 1. RCT (7) 2. Cluster RCT (1) 3. Quasi-experimental design (1) 4. Pre-post design (1) | 1. General medical practices (1)  2. VA health centre facility (1)  3. HIV/AIDS clinic (5) 4. City Seniors Centre (1) 5. Hospital (1)  6. University clinic (1) | 1. US (5) 2. UK (2) 3. Uganda (1) 4. Mozambique (1) 5. Botswana (1) | - Percentage female range:  25 – 92  - Ethnicity:  1. 90% white 2. 80% white  3. 47% white  4. 46% African American, 44% Hispanic  5. African (3)  - Veterans (1) |
| Fisher 2017 | Systematic review | To characterize peer support programs from around the world and their ability to promote sustained, complex health behaviours and, especially, such behaviours in diabetes prevention and management | 1. RCT (15)  2. CRT (2) 3. Other controlled design (1)  4. Pre-post design (1)  5. Pilot (1) | 1. AIDS clinics (1) 2. Clinical setting (1) | 1. Uganda (1) 2. Mozambique (1) 3. UK (2) 4. USA (2) 5. Canada (1) 6. Ireland (1) | Based in a socioeconomically disadvantaged area of London (1) |
| Dale 2008 | Systematic review | To assess the evidence for peer support telephone-delivered interventions involving verbal communication and aiming to improve health and health behaviours | RCT (2) | Not reported | 1. USA (1) 2. Australia (1) | 1. Over 65 years 2. aged 25 to 74 years |
| Lauckner 2016 | Scoping review | What is known from the existing literature about the key features and potential formats of community-based peer support initiatives for adults living with chronic conditions in rural settings? | Not reported | Rural, community-based peer support (13) | 1. USA (11) 2. Canada (1) | Women (4) |
| Merianos 2016 | Literature review | To examine the effectiveness of mentoring and peer-led interventions that includes a social support component to increase quality of life | 1. Controlled trial (2) 2. Non-comparative pre-post design (3) | 1. Online (1) 2. Camp (2) 3. School (1) | 1. Canada (1) 2. USA (2) 3. Australia (1) | - Age range (average):  7-37 years |
| Chronic kidney disease | | | | | | |
| Bennett 2018 | Literature review | To review patient-to-patient peer mentoring studies relevant to those with ESKD, receiving dialysis, and describe how mentoring may be positively implemented | 1. RCT (2) 2. Exploratory descriptive methods (2) | Haemodialysis centres (3) | 1. USA (2) 2. Taiwan (1) | Not reported |
| Diabetes | | | | | | |
| Cici 2018 | Systematic review | To collect evidence based on previous research result on the effect of peer group support on adult patients with Type 2 Diabetes Mellitus | 1. Non RCT 2. RCT with proportional stratified sampling 3. RCT (8)  4. Randomised Trial (2) 5. Partial randomised study  6. Quasi Experiment | Not reported | Not reported | Not reported |
| Dale 2012 | Systematic review | To address the need for rigorous systematic appraisal of the published evidence related to the impact and effectiveness of interventions that use peer support to improve the outcomes of adult patients with diabetes | 1. RCT (10)  2. Randomized comparative trials (4)  3. Non-randomized comparative study (1)  5. Before-and-after design (5)  6. Descriptive studies (2)  7. Feasibility study (1)  8. Case study (1) | Not reported | 1. USA (16) 2. UK (4) 3. Ireland (1) 4. Australia (1) 5. Netherlands (1) 6. Canada (1) | 1. American Indians ⁄ Alaska Natives 2. African American women  3. Spanish-speaking 4. African Americans  5. African American women;  6. Male  7. Aged 18–75; |
| Gatlin 2017 | Systematic review | Does diabetes self-management education taught by a peer educator improve clinical outcomes in individuals with T2DM when compared to routine care? | RCT (7) | Not reported | 1. UK (3) Argentina (1)  2. USA (2) 3. Australia (1) | - Age (range):  49.2 – 71.5  - Studies mostly comprised men  Ethnicity 1. Primarily non-Hispanic White sample (1)  2. Tailored to a specific [unspecified] ethnic group (2) |
| Kong 2020 | Meta‐analysis | To assess the effect of peer support intervention on diabetes distress in people with type 2 diabetes | RCT (13) | Not reported | 1. UK (2)  2. US (5) 3. Netherlands (1) 4. Argentina (1) 5. China (3) 6. Canada (1) | Age range (average)  50.2 – 67.8  Percentage Male range)  35.5 – 100 |
| Krishnamoorthy 2018 | Meta‐analysis | To assess the effectiveness of peer led intervention in improving the clinical outcomes of Diabetes mellitus and hypertension patients around the world. | Parallel arm individual or cluster RCT (29) | 1. Community (18);  2. Clinic (9);  3. Community and clinic (1);  4. Unclear (1) | 1. USA (12) 2. UK (3) 3. Ireland (1)  4. Vietnam (1) 5. Argentina (1) 6. Hong Kong (1) 7. Canada (1)  8. Austria (1) 9. Australia (1)  10. China (2) 11. Iran (3) 12. Philippines (1) 13. Mali (1) | - Age (average):  59.16  - Age (range):  50.2-68.8 |
| Qi 2015 | Meta‐analysis | To assess the effects of peer support at improving glycemic control in patients with type 2 diabetes | RCT (13) | 1. Public health clinics (1)  2. Outpatient clinic (1) 3. Veterans Affairs medical centre (1)  4. Unspecified practices (1) 5. Community-based (1) 6. Community health centres/ clinics (2)  7. Churches (1) | 1. USA (6)  2. Vietnam (1)  3. Ireland (1) | - Age range (average)  52.2 – 66.1  - Percentage female range:  44 – 100 |
| Tang 2011 | Literature review | To examine the diabetes-related health impact of volunteer-based peer support interventions in diabetes | 1 . RCT (2)  2. Pre-post design (1) | 1. At home, supported by Veterans Administration, VA (2) | 1. USA (2) 2. UK (1) | - Age range (average):  62 – 65.8  - Percentage female (range):  0 – 12  - Percentage white ethnic group:  79 – 95  1. Employed  2. 67% annual income < $30,000 (1)  3. 63% annual income < $30,000  [Data represents average across peer support and control condition] |
| HIV | | | | | | |
| Boucher 2020 | Systematic review | Does participation in a peer led self-management intervention affect adherence to ART among PLWH compared to participation in a comparison group? Furthermore, do peer-led self-management interventions affect other PROs for PLWH? | 1. RCT (12)  2. Quasi-experimental design (1) | 1. General Hospital & Health Centre  2. Online  3. The physiotherapy department of the public community health centre 4. Multi-site (2)  5. Community settings (2)  6. Infectious disease hospital units  7. HIV clinic  community-based  8. AIDS Clinical Trials Unit (2) | 1. USA (10) 2. Australia (1) 3. South Africa (1) 4. Spain (1) | 1. Women (1) 2. amaXhosa women (1) 3. Men who have sex with men (2) 4. African American or Puerto Rican populations with low socioeconomic status (1) |
| Somatic chronic illness | | | | | | |
| Kingod 2017 | Qualitative systematic review/qualitative evidence synthesis | To examine findings across qualitative studies to understand how individuals with chronic illness experience online peer-to-peer communities (which include both forums and interactive groups) and how these experiences influence daily life with illness. | Qualitative studies (13) | Not reported | Not reported | Not reported |

| **Study ID** | **Authors' definition of peer support** | **Review inclusion criteria** | **Review exclusion criteria** | **Comparator** | **Date limitation of search** | **Funding** |
| --- | --- | --- | --- | --- | --- | --- |
| Acquired brain injury, cerebral palsy, and spina bifida | | | | | | |
| Levy 2019 | Peer support is defined as support for a person with a chronic condition from someone with the same condition or similar circumstances | 1. Peer support interventions for adults (16 years of age or older) with acquired brain injury, cerebral palsy, or spina bifida  2. Measured community integration and/or QoL using validated scales, classifications, and/or measurement systems  3. Randomized controlled trials only 4. Only English-language publications | Conference abstracts and proceedings | 1. Self-efficacy training programme (1)  2. Non-mentored control group (1) | Jan 08 – Jun 18 | Toronto Rehab Foundation |
| Asthma | | | | | | |
| Kew 2017 | Peer support is a general term that may apply to many types of interventions for which the common factor is participation of a person or people similar to those for whom the intervention is provided. One concept analysis fully defined peer support as “the provision of emotional, appraisal and informational assistance by a created social network member who possesses experiential knowledge of a specific behaviour or stressor and similar characteristics as the target population” | 1. Parallel randomised controlled trials (RCTs)  2. Studies that used individual or cluster randomisation 3. Studies reported as full text, those published as abstract only and unpublished data studies that described inclusion criteria for asthma, such as confirmation by a physician or via spirometry 4. Adolescents as those between 10 and 19 years of age (or if the mean age of participants was between 10 and 19 years) 5. Studies that assessed an intervention delivered by peers or by lay people to adolescents with asthma 6. Peers as people who are not medically trained but are similar to the target population in terms of age, presence of an asthma diagnosis or diagnosis of a different long-term condition 7. Interventions delivered to individuals or groups of adolescents with asthma, irrespective of the mode of delivery 8. We included studies regardless of the aim of the intervention (e.g. improving self-esteem, improving medication adherence, providing asthma education). | 1. Cross-over studies owing to the likelihood of carryover effects 2. People with wheeze not associated with obstructive airways disease 3. Studies that enrolled adolescents with other long-term conditions, such as cystic fibrosis, unless the study authors presented results for participants with asthma separately 4. Interventions delivered by adult community health workers 5. Studies that used basic peer support itself as a minimal control for a more intensive intervention 6. Studies of interventions that involved multiple components other than the peer support or lay-led intervention unless the control group also received them | 1. Triple A Programme (peer support) alone (1) 2. Adult-led asthma camp (1) | Inception - present | NIHR, via Cochrane Infrastructure, Cochrane Programme Grant or Cochrane Incentive funding to the Cochrane Airways Group |
| Cancer | | | | | | |
| Campbell 2004 | Peers provide informational, emotional and practical support with the goal of helping patients better understand and cope with the disease | 1. Formative (e.g. needs assessments) and summative (e.g. outcome evaluations) studies  2. Peer support programs provided by volunteer cancer survivors to cancer patients  3. Published in English-language peer-reviewed journals. Only support programs which clearly articulated that health professionals had a facilitative non-directive role were included. | Programs directed at adolescents or caregivers were excluded | 1. Clients with breast cancer clinic patients not receiving peer support (1) 2. Non-users (1)  3. Waitlist controls (1)  4. Education only (1)  5. Routine care plus information (1)  6. Routine care plus nurse (1) 7. Usual care (2) | Jan 80 – Feb 02 | Not reported |
| Dunn 2003 | Peer support is defined as emotional support based on shared personal experience | 1. cancer patients were the target group for support, 2. the support program described was primarily for the delivery of peer support, 3. the article included either a qualitative or quantitative evaluation of the program | Not reported | Not reported | 1990 – Mar 01 | Not reported |
| Hoey 2008 | Peer support refers to support offered to people with cancer by people who have also experienced cancer | 1. written in English 2. described a specific program where peers provided direct support to people with cancer 3. peer had been diagnosed and/or treated for cancer 4. primary purpose of the program was to provide support to cancer patients. | 1. not a specific peer-support program 2. focused on children or adolescents 3. educational or therapeutic course run by professionals (including supportive expressive therapy) 4. not primarily focused on peer support, i.e., peer support was one of many components of the program 5. the peer support was for someone other than the person with cancer (e.g., carer or friend)  6. the paper did not describe the program in sufficient detail 7. how the peer provided support could not be determined 8. could not determine if the person giving support had experienced cancer 9. editorial or letter about a program  10. first-person account of an experience | 1. Non-program users/sites (3) 2. Other forms of support (1) 3. Unspecified control group (2)  4. Usual care (1) 5. Professional support (1) 6. Wait listed control (3) 7. Education or unspecified control (2) | 1980 – Apr-07 | Department of Health and Ageing, Commonwealth of Australia, administered through Cancer Australia |
| Lee 2018 | Peer support is a common form of social support because it provides patients with opportunities for experiential empathy. Peer-led supportive interventions (PSIs), in which individuals communicate and share experiences with others who have had similar personal experiences | 1. All included studies were RCTs or non-RCTs that examined the effects of supportive interventions led by the peers or partners of adults aged 18 years or older who had been diagnosed with cancer 2. these interventions were compared with healthcare professional–led care or usual care 3. English language | 1. They used group dynamics or a group-based format of peers with cancer 2. The peer-led interventions were supplemental to direct intervention from a healthcare professional 3. The patients with cancer were receiving hospice or palliative care 4. The peer-led support was a self-help group (participants were included in a group of individuals with similar illness and received support from those group members) or was Internet-based. | 1. no intervention  2. nurse-led cognitive-behavioural sexuality intervention, or educational materials  3. Usual care (5)  4. Wait-list followed by usual care (2)  5. American Cancer Society Reach to Recovery program  6. Brief telephone counselling with workbook  7. psychosocial intervention  8. Health education | 1997 – May 17 | National Research Foundation of Korea (NRF) grant funded by the Korean government (Ministry of Science and ICT) |
| Macvean 2008 | Peer support is a common form of social support and provides patients with the opportunity for experiential empathy | 1. a program where unpaid volunteers provided one-to-one support to people with cancer  2. Support could include emotional, psychological, peer, practical, referral and informational assistance  3. Assistance could be given to the person with cancer, or to this individual and their significant other, but not in group or course format  4. Provision of care could be through direct contact or telephone. | if the term ‘volunteer’ referred to voluntary participation in a research study or to a family member or friend acting as voluntary caregiver | 1. People at non-program sites 2. Non-users (2)  3. Other forms of peer support 4. Wait list 5. Usual care | Not reported – Apr-07 | Not reported |
| Meyer 2015 | Providing emotional or informational support to a patient who is at an earlier stage of treatment or recovery than the provider of peer support | 1. Peer-reviewed journal article reporting on an empirical study;  2. study describing one-to-one peer support programmes and/or interventions and the outcome of such interventions;  3. intervention/programme developed for cancer patients;  4. intervention delivered in person or by phone;  5. study conducted among adult cancer populations, that is, 18 years of age or older;  6. article published in English | 1. not an empirical study (e.g., reviews and study protocols) and/or not a peer reviewed publication (e.g., theses and dissertations) 2. article reporting on the evaluation of preventive interventions with peer educators 3. peer interventions developed for populations other than cancer patients themselves (e.g., for family members of cancer patients) 4. articles examining the effects of peer support groups and Internet one-to-one peer interventions (e.g., web-based message board) 5. peer support interventions for children/adolescents (i.e., below the age of 18) with cancer 6. article not published in English | Usual care (3) | May 07 – Jul 14 | Not reported |
| Walshe 2018 | Peer support involves people drawing on shared personal experience to provide knowledge, social interaction, emotional assistance or practical help, often in a way that is mutually beneficial | 1. Studies exploring the form, experience or impact of any form of peer support for people with cancer 2. Peer support has to include being supported by someone with personal experience of cancer within a ‘created’ social network network (this may be part of a wider or more complex intervention) 3. The population receiving peer support must include adults with a diagnosis of advanced/metastatic cancer 4. Primary research, any research design 5. Papers published after 2014 if the focus is on one-to-one support, after 2010 for other forms of peer support, to avoid duplicating existing reviews | 1.The question is on hypothetical rather than actual support 2. The focus is on peer support for cancer screening or prevention 3. The focus is on training people to be peer supporters 4. The support is provided by ‘embedded social networks’ (e.g. friends, neighbours), community volunteers, or health and social care professionals 5. The population are only those with early stage or curable cancer 6. The peer support is only provided to family carers, parents or children 7. Review papers  8. Papers in languages other than English | 1. Wait list (2) 2. [Unspecified] control condition  3. Moderated online support group | 2010 – Not reported | None |
| Hu 2019 | Peer support refers to the approach whereby individuals with the same disease or condition meet in order to exchange information, share experiences, and encourage or help each other to overcome difficulties | 1. English or Chinese language RCTs published in peer-reviewed journals or theses  2. study participants were diagnosed with breast cancer (including metastatic breast cancer and recurrent breast cancer)  3. peers had been diagnosed with breast cancer, and  4. the main purpose of the program was to support breast cancer patients. | 1. reviews, editorials, or letters about a program  2. intervention processes run by professionals  3. not a specific peer support intervention  (4) the peer support was targeted at other people besides breast cancer patients | 1. Waitlist (2)  2. Counsellor call (1) 3. Internet-based education (1) 4. Usual care (9);  5. “Idem” (1)  6. Moderated groups with a semi-structured (psychoeducational) format using asynchronous communication by social workers (1) | Inception – Jun 18 | National Natural Science Foundation of China |
| McCaughan 2017 | A form of peer support that consists of group members meeting face-to-face or communicating by telephone or via the Internet (including email and Facebook) for the purpose of sharing information and experiences and providing support on an issue or on topics of mutual interest. | 1. All randomised controlled trials (RCTs) assessing effects of online support groups on women with a diagnosis of breast cancer and those who have completed treatment for breast cancer 2. We included studies comparing online support groups with a usual care group, and studies comparing two or more types of online support groups (without a usual care group) 3. Included studies enrolled women with a diagnosis of breast cancer (any stage), disease free or not 4. Studies with mixed cancer populations and studies including partners were eligible for inclusion if they provided separate data for women with breast cancer 5. We included all types of treatment 6. All types of support groups involving more than two participants, offered via the Internet in the form of messaging (on a dedicated website or through email) or chat rooms, were eligible 7. We included both professional and user-led groups and combinations of these types of support 8. We compared online support groups against an inactive control intervention group (standard care or waitlist control) or against an active control intervention group (e.g. another form of psychological intervention) | Studies that evaluated a combination of face-to-face, telephone and online communication | 1. Information on a cancer‐related website 2. Bi‐weekly breast cancer newsletter by email 3. Moderated peer support condition | Not reported – 02 May 16 | HSC R&D Division of the Public Health Agency (Northern Ireland) |
| Cardiovascular disease | | | | | | |
| Clayton 2019 | Not reported | 1. were peer-reviewed (including grey literature)  2. were conducted within a CVD population  3. assessed an intervention in which social support was an outcome measure. | 1. were case studies, books, book chapters, or editorials  2. did not have pre–post measure of social support  3. were interventions conducted exclusively for individuals with congenital heart disease  4. were not published in English | 1. Usual care (3) 2. Usual care with shortened nurse practitioner led self-management education group session | Not reported | Michael Smith Foundation for Health Research Scholar Award |
| Jackson 2009 | Not reported | 1. adult participants with CHD (post-MI, coronary artery bypass graft, percutaneous coronary angioplasty, and angina) 2. Community-based non–health service-organized groups were included groups solely for people with CHD, including caregivers (people who undertake a substantive informal care role for a family member or friend) and partners, were also included | Hospital-based group treatment and therapy interventions or programs | Not applicable | None | Chief Scientist Office of the Scottish Executive |
| Parry 2010 | Peer support is a specific type of social support that includes appraisal, informational and emotional support. Informational support can increase knowledge, understanding and coping skills | 1. Included studies were published in English with specified intervention and control conditions | Non-randomized studies, dissertations and conference proceedings | Usual care (5) | Not specified – 2005 | Not reported |
| Small 2013 | A source of support, internal to a community, who share salient target population similarities (e.g. age, ethnicity, health concern, or stressor) and possess specific knowledge that is concrete, pragmatic and derived from personal experience rather than formal training | 1. RCTs  2. Adults (aged ≥ 18 years) with a diagnosis of vascular disease or long-term conditions associated with vascular disease, including: cerebrovascular disease, peripheral vascular disease, ischemic heart disease, stroke, heart failure, CKD, diabetes, and hypertension  3. Based on non-healthcare professional delivered telephone based self-management support | 1. If the intervention was delivered by a qualified or trainee health care professional;  2. If the calls were not supportive in content (i.e. reminder calls to assess medication compliance, involving one or two questions only);  3. If the telephone support was patient initiated only (i.e. patients called the support service). For inclusion, studies had to involve program-initiated calls, but could include patient-initiated calls alongside program-initiated ones;  4. If the intervention was home telemedicine (i.e. where the use of information technologies allow face-to-face contact through videoconferencing and may include the storage of clinical digital samples which are sent to the provider via electronic transmission such as email or via a telemedicine hub);  5. Non-English language publications;  6. Studies dealing with subcategories of vascular disease (e.g. Buerger’s disease and blood clotting disorders), which are not currently embedded within the routine management of vascular conditions, were excluded | 1. Usual care plus heart disease brochures (1) 2. Enhanced usual care consisting of an educational session plus support via nurse care manager (1) 3. Usual care (2) 4. Preoperative and postoperative education (1) 5. ‘Minimal intervention’ education pamphlets mailed to participants (1) | Not reported | Flexibility and Sustainability Funding (FSF) awarded to NS by the NIHR Greater Manchester CLAHRC awards committee |
| Chronic disease | | | | | | |
| Embuldeniya 2013 | Peer support is considered a unique type of social support provided by those who share characteristics with the person being supported and is intentionally fostered within formal interventions | 1. Studies were published in English  2. Interventions that featured individuals with a chronic disease and a structured peer support intervention led or co-led by a peer were included  3. Studies needed to feature qualitative methods | Quality appraisal criteria | Not applicable | Not reported | Not reported |
| Enriquez 2016 | Individuals living with the same chronic disease as the target population) as facilitators | 1. A peer-facilitated behavioural intervention had to have targeted a group of people living with a chronic disease who had been prescribed medications for said chronic disease by a physician, nurse practitioner, or physician’s assistant  2. To be included in this review, solely peers (i.e., meaning lay individuals living with the same chronic disease) delivered the intervention 3. Articles published in English | 1. Interventions that focused on enhancing medication adherence in diseases that require taking medications for a finite time frame were excluded 2. Reports of medication adherence interventions in chronic diseases that were facilitated by health care professionals or paraprofessionals were excluded 3. Interventions that focused strictly on enhancing healthy behaviours in chronic disease that did not involve taking medications (e.g., exercise, healthy eating) and hence did not include medication adherence as an outcome measure 4. Interventions where the peer was a proxy facilitator | 1. Usual care + nurse telephone support (1) 2. Nurse case management;  Usual care (6) 3. Pager messaging or peer support + pager messaging + pager messaging + usual care (1) | None – 31 Dec 14 | National Institute of Nursing Research |
| Fisher 2017 | Emotional, motivational and practical assistance provided by nonprofessionals for complex health behaviours | 1. Ongoing support from a nonprofessional 2. Assistance or consultation in applying management or behaviour change plans in daily life 3. Social and emotional support directed toward emotional status, wellbeing, or quality of life 4. Encouragement of recommended care | 1. Studies were excluded if the intervention was conducted by a professional. This was operationalized as post-baccalaureate training in a health profession  2. Addressed temporally isolated behaviours or single behaviours (e.g., mammography, vaccination) rather than complex behaviours extended over time 3. Was limited to a fixed number of group programs or classes or peers implementing a highly scripted information program. Group programs taught or facilitated by nonprofessionals are important strategies of health promotion but do not constitute ongoing PS for sustaining health behaviours of the sort the present review was intended to evaluate 4. Involved non professionals in roles limited to assisting others, such as in setting up rooms, distributing materials in classes, etc 5. Indicated PS as the outcome variable rather than the independent variable, it being the intent of this review to assess the effects of PS 6. No statistical tests of significance of changes observed, rendering indistinguishable reports of changes versus nonsignificant changes 7. Control or comparison conditions that provided a substantial amount of social or PS that may have masked or obscured the effects of social/peer support. This included, e.g., studies of group interventions in which all conditions included encouragement of social support among group members. However, given that PS is intrinsic to almost any group intervention and thus to avoid shrinking the pool of papers substantially, we retained several papers, (e.g., [38]) that included support in all conditions, but which focused evaluation on additional PS in an experimental condition. | 1. Usual care (7) 2. Usual care support groups (1) 3. Standardized diabetes care (1) 4. Telecare support by specialist nurses (1) 5. Enhanced usual care of 1:1 consultation with care manager, self-care materials, optional phone or face-to-face follow-up  6. Week-long rehabilitation program (1) 7. Waitlist control (1) 8. Information on surgery recovery (1) 9. Internet-based education | 01 Jan 00 – 15 Aug 11 | Not reported |
| Dale 2008 | The most comprehensive definition of peer support describes a peer as a created source of support, internal to a community, who shares salient target population similarities (e.g. age, ethnicity, health concern or stressor) and possesses specific knowledge that is concrete, pragmatic and derived from personal experience rather than formal training | 1. Randomised controlled trials (RCTs) 2. compared peer support telephone calls with other types of intervention and/or with usual care or compared different models (e.g. different levels of training) of peer support telephone calls with each other and/or with usual care 3. We included people living with acute or long-term illness, carers of people with acute or long-term illness, parents, people with psychological symptoms, and people requiring screening or who had any other health and well-being related concerns. We included peer support telephone calls (of any duration) based on verbal communication, in which the peer is someone selected to provide support because they have similar or relevant health experience | 1. data on the peer telephone element could not be extracted 2. they were not RCTs 3. the peer component was not delivered by telephone  4. peers were not used for intervention delivery 5. the intervention was facilitated by non-peers | 1. Unspecified usual care  2. Usual care consisting of discharge instructions provided by the clinical nurse. Discharge instructions included a review of medications, diet, physical activity, symptom management and follow-up appointments | Inception – Dec 07 | Department of Health Systematic Review Fund, UK |
| Lauckner 2016 | For the purposes of this article, refers to support for a person with a chronic condition from someone with the same condition or similar circumstances | 1. interventions/programs (rather than opinion pieces) 2. the years 2000–January 2014 (to ensure currency) 3. English only (due to language limitations of the research group) 4. adults (18 years or older) 5. explicit involvement of peers who work with people who have a chronic condition the peer is familiar with 6. community-based (rather than hospital-based) with an emphasis on community involvement (rather than medical management) 7. explicit reference to being located in rural settings | 1. professional-led initiatives with no focus on the development of peer supports 2. initiatives that only focus on friendship development without reference to community involvement  3. initiatives that focus on caregivers of people with chronic conditions 4. involvement of lay leaders who do not have lived experience of chronic condition | Not reported | Inception – Jul 13 | Not reported |
| Merianos 2016 | Not reported | 1. studies published between January 1, 2001 and June 1, 2014  2. studies published in English  3. studies published in peer-reviewed journals  4. studies that included adolescents from 12 to 17 years of age  5. community-based psychosocial programs (i.e., mentoring and peer led) provided for adolescents with chronic illnesses  6. studies that included a social support component to increase quality of life outcomes  7. studies with a health education focus  8. studies that utilized a quantitative methods component 9. Studies that had strong methodological designs that had either a comparison group or pre- and post-test evaluations for treatments and control groups  10. studies from all geographical locations | Not reported | 1. Unspecified control group (2) | 01 Jan 01 – 01 Jun 14 | Not reported |
| Chronic kidney disease | | | | | | |
| Bennett 2018 | The patient-to-patient peer relationship is one that is non-hierarchical and reciprocal with peers sharing similar experiences and knowledge with others who have undergone similar challenges | 1. Studies that had implications for dialysis 2. Studies containing quantitative and qualitative methods, or a combination of both | 1. Studies addressing CKD only 2. Non-English studies 3. Studies published before the year 2000 4. Studies focusing on children and/or adolescents, including those focusing on kidney camps as an intervention | 1. Printed materials (1) 2. Unspecified control (2) | 2000 – Not reported | Not reported |
| Diabetes | | | | | | |
| Dale 2012 | ‘Support from a person who has experiential knowledge of a specific behaviour or stressor and similar characteristics as the target population’ | 1. published in English 2. described a specific programme which included peers providing support to adults with diabetes  3. all subjects (patients) were diagnosed and being treated for diabetes 4. study designs included randomized or quasi-randomized controlled trials, controlled clinical trials, before-and-after studies, interrupted time series, descriptive studies or case studies  5. interventions were aimed at improving the care or management of diabetes. | Not reported | 1. Usual care (5) 2. Usual care or Diabetes specialist nurse (1) 3. Nurse case management (1) 4. Specialist health professional training on self-management (1) 5. Unclear (5) 6. Information only or Personal self-management coach (1) | 1966 – Dec-11 | None |
| Tang 2011 | Dennis defines peer support as “the provision of emotional, appraisal, and informational assistance by a created social network member who possesses experiential knowledge of a specific behaviour or stressor and similar characteristics as the target population, to address a health-related issue of a potentially or actually stressed focal person.” | 1. Involving volunteer peer supporters. We considered peer supporters to be volunteers if they were not full- or part-time employees of the clinics, organizations, or universities in which they served. We did include programs in which peer supporters received a stipend or honorarium used to offset the costs of participation (e.g., transportation and childcare);  2. describe an intervention focused on self-management rather than prevention;  3. include an explicit training component for peer supporters;  4. report clinical, behavioural, knowledge, and/or psychosocial outcomes;  5. use an RCT or quasi-experimental (e.g., pre-/post-or case-comparison) study design | Studies involving family members as peer supporters were excluded | 1. Standard care, individual 15- to 20-minute appointment with dietitian 2. 90-minute education session and assignment to a nurse case manager | 1990 – Nov 10 | 1. Peers for Progress  2. The American Association of Family Physicians Foundation |
| Cici 2018 | Peer support is defined as the support from someone who has knowledge of experience about a particular behaviour or stressor with the characteristics similar to the population target | 1. Peer Group Support Program  2. All participants were patients diagnosed with Type 2 Diabetes Mellitus 3. Patient age 18-75 years  4. Quantitative studies with Non and Randomized Controlled Trial, Randomized Clinical Trial, Partial Randomized Study designs | 1. Patient age <18 years or >75 years  2. Not yet diagnosed with Diabetes Mellitus  3. Type 1 Diabetes Mellitus  4. Qualitative Studies | Not reported | 2012 – 2017 | Not reported |
| Gatlin 2017 | Peer education was defined as education delivered by a trained individual with the same illness as the person they were educating | 1. English-language RCTs in  peer-reviewed journals  2. compared a peer education intervention to a usual care control group  3. measured objective clinical or  psychosocial outcomes, such as HbA1c, body mass index, blood  pressure, lipid levels, self-efficacy scores, depression or levels of  knowledge at both pre- and postintervention | The primary element of peer education used to include studies was the presence of a diabetes self-management knowledge measure. Studies that did not meet this definition were not included | 1. Specialist health professional (control group) 2. Usual care from a dietitian  3. Structured diabetes education course only by professional educators 4. Continuation of standard diabetes care 5. Patients in the control group individually attended standard diabetes education sessions 6. Usual care 7. Usual education only | 2006 – 2016 | Not reported |
| Kong 2020 | Peer support refers to provision of support by an individual who has knowledge from his own experiences with a chronic condition (Patil et al., 2016) | 1. participants (diagnosed with type 2 diabetes or comorbidity of type 2 diabetes and other chronic conditions)  2. intervention (peer support provided by people with diabetes or affected by diabetes [e.g., family or caregiver])  3. control (usual care; both the intervention group and control group received similar baseline care)  4. outcome (diabetes distress was assessed by validated measures; the outcome was compared at baseline and a predefined follow‐up time points)  5. study design (RCTs). | 1. studies without baseline values and 2. use of additional interventions provided by health professionals that could influence outcomes. | 1. Single call 2. Care from nurses 3. Usual care 4. Structured education 5. Diabetes education 6. Integrated care 7. Support call 8. Usual education (2) 9. Usual care 10. Education from health care providers | Inception – 30 Jun 18 | Training program, Chongqing Federation of Social Science Circles |
| Qi 2015 | Peer support has been defined as ‘support from a person who possesses experiential knowledge of a specific behaviour or stressor and similar characteristics as the target population | 1. RCTs, because this study design has maximum validity and causal inference [26]  2. adults (aged ≥18 years) diagnosed with T2DM  3. studies that reported HbA1c levels, which is an index of the mean blood glucose concentration of the preceding 8–12 weeks and is the recommended index for evaluation of glycaemic control of diabetes  4. peer support represented the majority of the interventions; 5. compared with a usual or routine care group. | 1. the intervention was delivered by a health care professional  2. the intervention did not involve direct contact between peer supporters and patient or was unclear  3. non-English language publications. | 1. Usual care unspecified (2) 2. UC access to a nutritionist and diabetes educator through referral from their primary care clinician (1) 3. UC follow-up (1) 4. UC regular recall of patients every three to six months with an annual audit of risk factors (1) 5. UC asked to maintain normal daily routine (1) 6. UC one-to-one patient education (1) 7. UC educational pamphlets (1) | Not reported – Dec 14 | 1. Chongqing Health and Family Planning Commission 2. Chongqing Health Bureau |
| Krishnamoorthy 2018 | A concept analysis has defined the peer support as the provision of informational, appraisal and emotional assistance by separate and created social network members who possess experiential knowledge regarding specific behaviour and similar in characteristics to that of the target population. We have defined the peers as the people who were not trained medical professionals but similar to the tar-get population in terms of presence of diabetes mellitus or hypertension diagnosis. | 1. parallel arm individual or cluster randomized controlled trials (RCT) 2. Studies reported as full text 3. adults aged more than 18 years who were already diagnosed with type 2 DM or HTN  4. studies that assessed the effectiveness of intervention provided by peers of diabetes or hypertension patients 5. trials irrespective of the mode of delivery of interventions which can be individual or group-based intervention delivered face-to-face or via technology such as mobile calls 6. Studies assessing effectiveness of peer group intervention on diabetes patients and reporting the HbA1C level, effectiveness on hypertension patients and reporting the SBP values irrespective of the primary objective of the interventions 7. Studies irrespective of the baseline HbA1C levels and SBP levels | 1. Cross over studies published with only abstract or unpublished data 2. Studies conducted among participants with co-existing DM and HTN unless the results were presented separately for DM and HTN 3. studies which had intervention provided by other counsellors like community health workers | 1. Standard care (22) 2. Unspecified other intervention (4) | Inception – May 18 | None |
| HIV | | | | | | |
| Boucher 2020 | Support provided by those who share characteristics with the person being supported and is intentionally fostered within formal interventions | 1. Randomized controlled trials, non-randomized quasi-experimental studies 2. Participants of any age who were living with HIV 3. Any peer-led self-management intervention, which could also be co-led by a peer and a professional/non-peer 4. Usual care, health education/information/promotion, or intervention led by professional/non-peer (if a study had multiple comparison groups the one most similar to usual care was chosen) 5. Primary Adherence to highly active antiretroviral therapy/combination antiretroviral therapy/antiretroviral therapy. All types of adherence measures were included (e.g. self-report, physician or nurse report, pharmacy prescription logs, pill counts, directly observed therapy, electronic drug monitors, and antiretroviral drug concentration). Secondary Patient-reported outcomes (PROs), including various clinical and psychosocial outcomes (e.g. self-efficacy, health behaviours, symptoms, and health status measures). Our outcome list was intentionally broad to allow the addition of other PROs that emerged during the literature scan. | 1. Qualitative studies  2. intervention delivered before 1996 3. No people living with HIV  4. data for people with HIV not separate from people without HIV  5. Interventions focused only on providing health education, information, or promotion  6. No comparison group 7. Any outcome not measuring adherence or not considered a PRO | 1. Standard care: included educational efforts afforded to them as part of their usual treatment 2. Intervention led by graduate student (non-peers): time and contact-matched Healthy Eating comparison intervention and received 2 workbooks 3. Intervention led by educators (non-peers): provided didactic instruction on safer sex and safer drug use, contact time was comparable, including in-person and telephone sessions 4. Usual care: any other pharmacologic and non-pharmacologic treatments for chronic pain provided by their clinicians and not related to the study 5. Usual care (no information provided on what this involved) 6. Health information only: provided with educational workbooks 7. Intervention not including peer mentoring component: eight documentary or self-help video and facilitated discussion group sessions, nearly equal attention and controlled for experimental demand 8. Intervention led by a health professional: received the same psychoeducational intervention but led by a “physician or pharmacist with extensive knowledge about HIV”);  Standard care: given social and mental health referrals when requested 9. Usual care: included the HAART Protocol, a clinic-based program designed to provide education regarding HAART and adherence 10. Health information only: received a copy of HIV Symptom Management Strategies: A Manual for People Living with HIV/AIDS 11. Health information only: received written self-help materials for smoking cessation 12. Intervention not including peer-led component: consisted of one panel presentation that provided safer sex information, modelled after community forums | 18 Jun 1905 – 11 Mar 18 | 1. Canadian Institutes of Health Research (CIHR) Doctoral Research Award: Priority Announcement—HIV/AIDS 2. CIHR-Ontario HIV Treatment Network New Investigator Award |
| Somatic chronic illness | | | | | | |
| Kingod 2017 | Linking people with the same illness and similar characteristics to enable them to share knowledge and experience | 1. qualitative studies of online peer-to-peer support communities for adults with somatic chronic illnesses 2. peer-reviewed; qualitative studies 3. in English 4. pertained only to adults | 1. interactive patient-to-patient web-based solutions;  2. social support for adults with chronic illness;  3. peer-to-peer interactive online groups, forums, or communities | Not reported | Not reported – Oct 15 | 1. The Innovation Fund Denmark, and the Diabetes Management Research, Health Promotion Research, Steno Diabetes Center, Denmark 2. The European Research Council Starting Grant “The Vitality of Disease—Quality of Life in the Making” |
