# Supplementary material for: Peer support for people with chronic conditions: a systematic review of reviews
Source: BMC Health Serv Res. 2022 Mar 31;22:427. doi: 10.1186/s12913-022-07816-7 (PMC8973527; doi:10.1186/s12913-022-07816-7)
Supplement: Supplementary file 5 — Additional file 5. [file 12913_2022_7816_MOESM5_ESM.docx]

**Additional file 5** .docx; Training description

| Training content and methods | Reviews | |
| --- | --- | --- |
|  | Number | Percentage |
| Delivery methods |  |  |
| Role-play | 3 | 10% |
| Discussion | 2 | 6% |
| Observation | 2 | 6% |
| Didactic material | 2 | 6% |
| Practicing delivering content | 2 | 6% |
| Working in pairs | 1 | 3% |
| Lectures | 1 | 3% |
| Readings | 1 | 3% |
| Online education modules | 1 | 3% |
| Experiential learning | 1 | 3% |
| Modelling | 1 | 3% |
| Counselling skills |  |  |
| Encouraging behaviour change | 4 | 13% |
| Empowerment, motivational interviewing | 2 | 6% |
| Empathetic communication | 1 | 3% |
| Cessation counselling | 1 | 3% |
| Trauma recovery | 1 | 3% |
| Cognitive and emotional aspects | 1 | 3% |
| Positive thinking | 1 | 3% |
| Negative affective state assessment | 1 | 3% |
| Communication skills |  |  |
| Communication skills | 7 | 23% |
| Listening skills/ active listening | 4 | 13% |
| Telephone support skills | 2 | 6% |
| Interviewing skills | 1 | 3% |
| Condition and treatment information and adherence | | |
| Condition/ treatment knowledge | 5 | 16% |
| Review clinical results and create an action plan | 1 | 3% |
| Adherence support skills | 1 | 3% |
| Barriers to adherence | 1 | 3% |
| Meta-competency and safety |  |  |
| Defining what is/isn't mentoring | 1 | 3% |
| Review safety issues and how to handle crisis situations | 1 | 3% |
| Understand documentation responsibilities | 1 | 3% |
| Research ethics and conduct | 1 | 3% |
| Social skills and story sharing |  |  |
| Psychosocial issues | 3 | 10% |
| Provision of social support | 1 | 3% |
| Trained to share surgery experiences | 1 | 3% |
| Theory |  |  |
| Intervention theory and principles | 3 | 10% |
| Adult learning principles | 1 | 3% |
| Using a person-centred approach | 1 | 3% |
| Culture and religion |  |  |
| Spirituality discussion with Chaplaincy | 1 | 3% |
| Cultural sensitivity | 1 | 3% |
| Physical training |  |  |
| Functional exercises | 1 | 3% |
| Physical activity training | 1 | 3% |
| Facilitation skills |  |  |
| How to structure and run sessions | 1 | 3% |
| Community resources |  |  |
| Community resources | 1 | 3% |
